# Social media reveal ecoregional variation in how weather influences visitor behavior in U.S. National Park Service units

**DOI:** 10.1038/s41598-021-82145-z

**Published:** 2021-01-28

**Authors:** Emily J. Wilkins, Peter D. Howe, Jordan W. Smith

**Affiliations:** 1 Department of Environment and Society, Utah State University, Logan, UT USA; 2 Institute of Outdoor Recreation and Tourism, Utah State University, Logan, UT USA

**Keywords:** Psychology and behaviour, Environmental social sciences

## Abstract

Daily weather affects total visitation to parks and protected areas, as well as visitors’ experiences. However, it is unknown if and how visitors change their spatial behavior within a park due to daily weather conditions. We investigated the impact of daily maximum temperature and precipitation on summer visitation patterns within 110 U.S. National Park Service units. We connected 489,061 geotagged Flickr photos to daily weather, as well as visitors’ elevation and distance to amenities (i.e., roads, waterbodies, parking areas, and buildings). We compared visitor behavior on cold, average, and hot days, and on days with precipitation compared to days without precipitation, across fourteen ecoregions within the continental U.S. Our results suggest daily weather impacts where visitors go within parks, and the effect of weather differs substantially by ecoregion. In most ecoregions, visitors stayed closer to infrastructure on rainy days. Temperature also affects visitors’ spatial behavior within parks, but there was not a consistent trend across ecoregions. Importantly, parks in some ecoregions contain more microclimates than others, which may allow visitors to adapt to unfavorable conditions. These findings suggest visitors’ spatial behavior in parks may change in the future due to the increasing frequency of hot summer days.

## Introduction

Climate change poses risks to ecosystems within parks and protected areas as well as the outdoor recreation opportunities they provide^1–3^. Visitation will likely change at most parks as temperatures continue to rise, extreme heat events become more common, and precipitation becomes more variable^3–5^. To date, projected impacts to visitation in response to warming temperatures and extreme heat events have only been studied at the scale of whole park units^4,6^; we are unaware of any research examining how the spatial patterns of visitation may change within parks. Understanding how visitation patterns may change within a park due to weather can help park managers plan and prepare for managing visitor flows, both on a daily scale and when thinking about future climate change.

The overall objective of this study is to explore how the spatial behavior of visitors to U.S. parks changes during the summer in response to temperature and precipitation. Visitors’ spatial behavior captures where individuals choose to go during their park visit. Outdoor recreationists in parks make sovereign decisions about which trails to hike, which rivers to float, and which scenic overlooks to stop at, among many other decisions affecting the location of where outdoor recreation occurs^7^. All of these decisions are influenced, to varying degrees, by the weather. This research quantifies how, and to what extent, the weather influences park visitors’ spatial behavior during the summer. We focus on summer because the influence of weather on the spatial patterns of visitation likely differ by season, and because visitation-related management challenges are most often experienced in the summer, when visitation tends to be highest^8^.

We focus on two measures of visitors’ spatial behavior: the elevation of an outdoor recreation trip and the distances of that trip from roads, waterbodies, parking areas, and buildings. We test the hypotheses that visitors may be more likely to visit higher locations and stay closer to roads, waterbodies, parking areas, and buildings on extremely hot days, particularly in the warmest ecoregions. We hypothesize this because previous research shows there is a threshold that visitors consider too hot in parks, which may make visitors more likely to stay near infrastructure or seek cooler temperatures at higher elevations^6,9^. On days with high precipitation, we expect that visitors will stay at lower elevations and be closer to roads, parking areas, and buildings.

**Figure 1:**
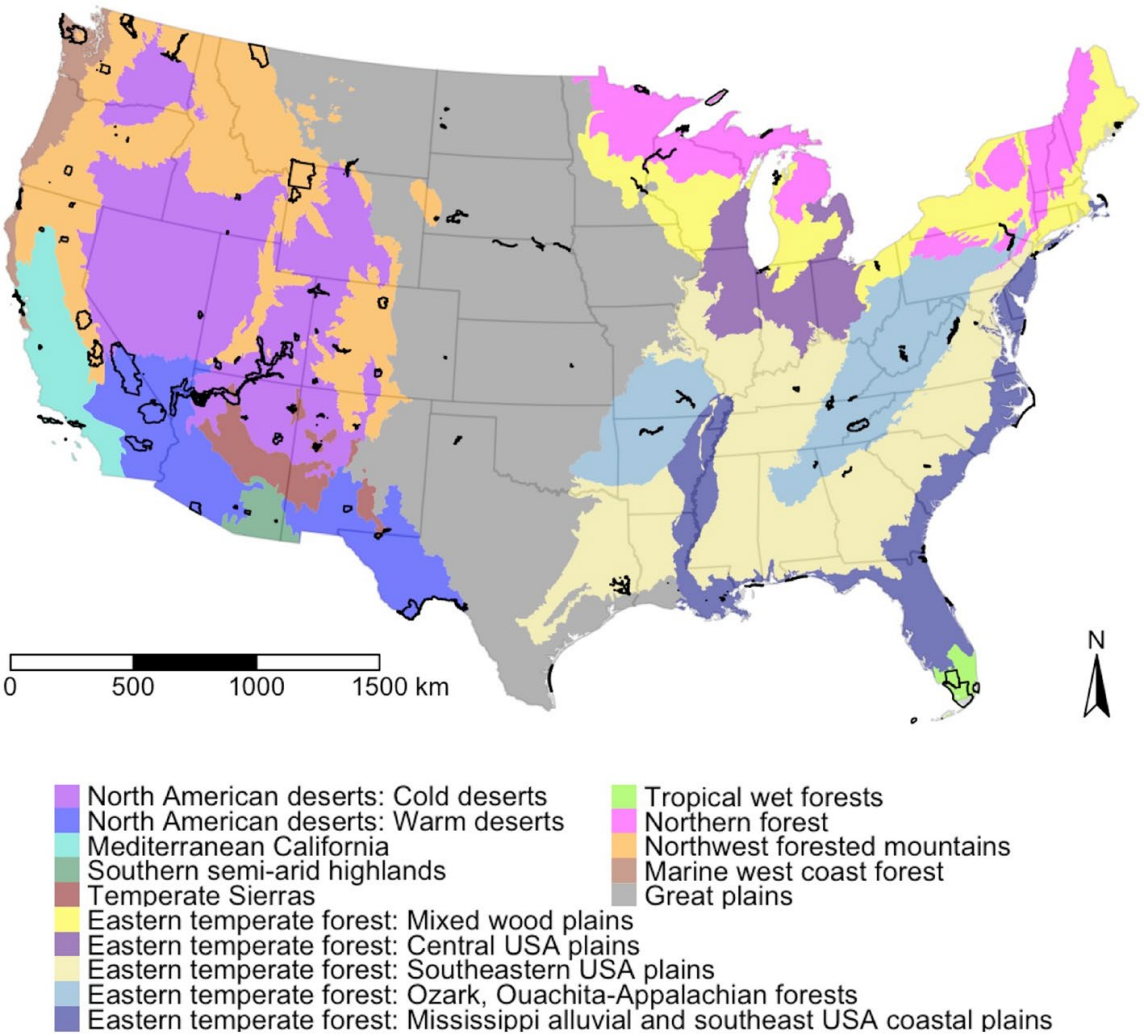
Locations of the 110 NPS units used in this study and continental U.S. ecoregions used to categorize parks. Figure created in R version 3.6.1 (www.r-project.org) with the tmap package^12^.

To test these hypotheses, we used geotagged social media to understand exact dates and locations of visits within 110 U.S. National Park Service (NPS) units. NPS units include national parks, national recreation areas, national monuments, and national seashores, among others; these are all considered different designations of parks^10^. Because of the geographic diversity of NPS units, the influence of weather on visitor behavior is likely to be highly variable. Warmer than average temperatures may cause visitors to travel farther from roads in relatively cool climates, but may cause visitors to stay closer to roads in hot climates. To account for this variability, we examine the proposition that the impact of weather on visitors’ spatial patterns within parks varies by ecoregion. Ecoregions represent areas in North America where the ecosystems are generally similar^11^. Figure 1 shows the categories of ecoregions used in this study; it also shows the 110 NPS units in this study.

We used geotagged social media from Flickr to understand spatial patterns of visitation given the fine spatial and temporal resolution of these data. Flickr is a photo-sharing application that has been previously used to understand park visitation and spatial patterns of visitors in parks^13^. We connected the dates and locations of posts to daily weather data at each place and time, and compared spatial distributions of visitors on cold, average, and hot days, as well as on days with precipitation compared to no precipitation. Our work is informed by both the growing body of research examining the influence of weather on outdoor recreation, as well as the literature on using social media data to understand park visitors^4,6,13,14^.

## The impact of weather on outdoor recreation

Outdoor recreationists often select their destinations and the timing of their trips based on the climate^15^. Once on-site, weather influences the types of activities chosen, the length of stays, and the amount of satisfaction obtained^16^. However, tourists’ sensitivities to and preferences for weather differ depending on the climate of their destination^17^. For instance, tourists in mountain areas or urban areas believe the ideal temperature is lower than the ideal temperature desired by beach tourists^18,19^. There is substantial variation found in the literature for optimal temperatures and thresholds for outdoor recreation, largely because outdoor recreation settings and the activities they support vary widely, and many studies tend to be focused on one or two specific settings^20,21^. For example, precipitation was found to be negatively correlated with summer visitation to a forested and beach park in Canada, and temperature positively correlated with visitation, up to a threshold of 33 °C, after which visitation declined^14^. A different study in five desert U.S. national parks found visitation declined at three parks once a threshold of 25 °C was reached, while two parks did not exhibit a temperature threshold^6^. We utilize nationwide visitation and weather data to analyze the impact of daily weather on the spatial behavior of visitors across multiple settings.

Changing temperature and precipitation patterns are likely to directly impact both the supply of and demand for outdoor recreation opportunities, although the impacts will also differ by activity and geographic region^3,22^. For example, previous research has found the impact of monthly weather averages on visitation to Australian parks varied by climate region^23^. Increased temperatures due to climate change have already expanded the length of the peak season in U.S. national parks^24^. Warmer than average temperatures generally equate to longer seasons in which individuals can participate in warm-weather recreation activities^1^. However, the ways in which weather impacts park visitation is likely to be dependent upon the geographic features of particular parks. Some outdoor recreation destinations may see visitation decline after reaching a certain temperature threshold (e.g., 25–33 °C), while parks with a greater number of different microclimates accessible to visitors (e.g., mountain parks or those with deep canyons) may continue to experience visitation increases above the threshold^6^.

Most studies to date have not taken into account different microclimates within a single destination. For example, Rutty and Scott^25^ found that coastal tourism areas contained varying microclimates, with thermal conditions differing up to 4 °C at various areas of a particular resort. Although some outdoor recreation destinations may appear “too hot” under altered climatic conditions^4^, it is unknown whether visitors may adapt by visiting different areas within a park (e.g., higher altitudes or near bodies of water). By joining the location and date of social media posts with historical weather data, we provide the first high-resolution understanding of how temperature and precipitation impact the spatial behaviors of outdoor recreationists within parks in the U.S.

## Using social media data in parks

Over the last decade, researchers have found social media data to be helpful to inform outdoor recreation management in parks and protected areas^13,26,27^. Social media can be used as a relatively accurate estimation of visitation to parks and protected areas at annual and monthly scales^28–30^. For example, social media from Flickr was found to be useful to discern monthly trends in visitation to national parks in the western U.S.^29^. Although many land management agencies in the U.S. estimate visitation through surveys, administrative data, and traffic counters^31^, social media data are unique in that they allow for visitation estimates at fine spatial and temporal resolutions. The NPS only produces visitation estimates at the monthly scale^31^, whereas social media data can show temporal trends in visitation at the hourly resolution^32,33^. This is because the timestamp that the photo was taken, and the geographical coordinates of the photo, are recorded in metadata automatically recorded by and stored on individuals’ smartphones^34^. For instance, one study used multiple years of geotagged Flickr data to understand trends in what time of day, and what day of the week, people tend to visit a national park in Spain^32^. Additionally, geographic coordinates of posts are typically accurate within 5 m if photos are taken with a GPS-enabled device^35^, making the spatial resolution higher than other sources of visitation data.

Researchers have also leveraged the spatial specificity of geotags to show trends in where visitors go within parks and protected areas^32,36–38^. By mapping social media along with other geospatial data, researchers can better understand what factors relate to visitor demand within a park^36,39,40^. For example, previous research has concluded the spatial patterns of Flickr posts in parks differ by season, and the presence of trails was the most important factor predicting Flickr photos in the summer in national parks^36^. The resolution of geotagged social media can be leveraged to understand how visitation patterns relate to infrastructure, like trails and roads, as well as environmental factors like weather.

## Results

### Correlations between flickr data and NPS-reported visitation

The correlation between Flickr Photo-user-days (PUDs) and NPS-reported visitation across 108 units was R_s_ = 0.707 (*n* = 108, p < 0.001). At the monthly scale, the correlation was R_s_ = 0.709 (*n* = 540, p < 0.001). These data are summed from 2006 to 2018 and include the months of May–September. This correlation is similar to other studies comparing social media posts in parks to other sources of visitation data^27^. Thus, results suggest geotagged Flickr data are a useful proxy for summer visitation in NPS units.

### Descriptive statistics

Table 1 shows all the means and standard deviations by ecoregion for daily maximum temperature at the visitor centers and Flickr points, daily precipitation at the visitor centers and Flickr points, and elevation at the visitor centers and Flickr points. Mean maximum daily temperature at visitor centers was highest in the warm desert ecoregion (37.1 °C) and lowest in the marine west coast forest ecoregion (22.5 °C). Mean daily precipitation at visitor centers was highest in the tropical wet forest ecoregion (6.3 mm) and lowest in the Mediterranean California ecoregion (0.1 mm). Overall, there was not much variation in the amount of daily precipitation at visitor centers compared to Flickr points. Elevation at visitor centers was highest for the cold deserts ecoregion (1829.0 m), and highest for Flickr points in the Northwest forested mountains ecoregion (1999.2 m). Flickr points in the Northwest forested mountains ecoregion had the largest standard deviation for elevation, indicating this ecoregion has the largest range of elevations visitors frequent. Elevation was lowest in the tropical wet forests ecoregion (1.2 m at the visitor centers, and 1.1 m at Flickr points).

**Table 1:** Means and standard deviations (in parenthesis) for all weather data and elevation by ecoregion.

Ecoregion	Flickr *n*	Max. temp at visitor centers (°C)	Max. temp at Flickr post (°C)	Precip. at visitor centers (mm)	Precip. at Flickr post (mm)	Elevation at visitor centers (m)	Elevation at Flickr post (m)
Warm deserts	25,784	37.1 (6.6)	35.2 (7.2)	0.3 (2.0)	0.3 (2.3)	478.3 (403.1)	722.2 (560.0)
Southern semi-arid highlands	1258	33.5 (4.6)	33.1 (5.7)	1.2 (3.5)	1.2 (3.7)	1088.9 (284.1)	1100.6 (479.3)
Tropical wet forests	2157	32.3 (1.6)	32.4 (1.6)	6.3 (10.3)	6.9 (12.7)	1.2 (0.4)	1.1 (0.9)
Southeastern USA plains	1391	29.6 (3.8)	29.5 (3.8)	3.9 (9.2)	3.9 (9.1)	210.4 (88.4)	197.9 (89.7)
Temperate Sierras	797	29.5 (4.9)	29.3 (5.5)	1.3 (4.8)	1.3 (4.7)	1506.5 (197.5)	1521.3 (350.2)
Mississippi alluvial and southeast USA coastal plains	18,337	27.6 (4.2)	27.8 (4.3)	3.5 (10.8)	3.2 (9.9)	5.1 (3.8)	3.7 (7.0)
Cold deserts	86,804	27.3 (6.1)	27.4 (6.0)	1.1 (3.1)	1.0 (3.1)	1829.0 (467.1)	1830.8 (501.7)
Ozark, Ouachita-Appalachian forests	17,830	27.1 (3.9)	25.3 (4.6)	4.1 (8.3)	4.6 (8.9)	387.0 (106.2)	770.3 (492.3)
Great plains	24,901	26.3 (5.0)	26.3 (5.0)	2.6 (6.9)	2.8 (7.4)	375.3 (241.0)	385.2 (258.0)
Mixed wood plains	14,228	24.1 (4.2)	23.8 (4.3)	3.1 (7.6)	3.3 (7.9)	99.0 (86.8)	172.5 (128.8)
Northern forest	6035	24.0 (4.2)	24.0 (4.2)	3.1 (7.7)	3.0 (7.9)	265.6 (93.3)	211.1 (47.0)
Northwest forested mountains	209,173	23.7 (6.7)	21.0 (6.0)	0.9 (2.8)	1.0 (3.0)	1606.8 (685.1)	1999.2 (770.6)
Mediterranean California	76,508	23.0 (4.3)	22.5 (4.2)	0.1 (1.2)	0.1 (1.3)	77.7 (63.3)	82.9 (137.6)
Marine west coast forest	3858	22.5 (3.2)	21.7 (3.4)	0.7 (2.8)	0.7 (2.7)	47.5 (0.0)	97.1 (126.1)

Values represent data from May to September.

Table 2 shows the means and standard deviations by ecoregion for the distance from each Flickr point to the nearest road, waterbody, parking area, and building. Mean distance to roads ranged from 9.3 m (Southeastern USA plains) to 165.2 m (temperate Sierras). Across all ecoregions, the mean distance to roads was 63.0 m, and the median distance to a road was 10.9 m. This indicates many visitors to NPS units stay very close to roads in the summer. In most ecoregions, visitors were farther from buildings and designated parking areas compared to roads. These results suggest many visitors may take photos from their cars, or from pullout areas on the side of roads.

**Table 2:** Means and standard deviations (in parenthesis) for all distance measures by ecoregion.

Ecoregion	Flickr *n*	Dist. to road (m)	Dist. to water (m)	Dist. to parking (m)	Dist. to building (m)
Warm deserts	25,784	83.9 (279.6)	3697.9 (9165.7)	1181.9 (4547.2)	462.9 (1131.6)
Southern semi-arid highlands	1258	26.0 (66.2)	355.4 (587.0)	347.0 (944.7)	402.7 (828.2)
Tropical wet forests	2157	120.4 (401.7)	319.9 (643.9)	666.4 (1216.9)	452.0 (1134.9)
Southeastern USA plains	1391	9.3 (17.2)	145.5 (268.7)	552.3 (1331.9)	174.2 (447.1)
Temperate Sierras	797	165.2 (287.4)	5829.1 (2924.4)	626.9 (1557.0)	612.4 (1586.6)
Mississippi alluvial and southeast USA coastal plains	18,337	161.7 (787.5)	73.1 (108.7)	594.2 (1377.0)	102.2 (222.1)
Cold deserts	86,804	72.3 (351.4)	941.8 (1918.5)	549.2 (1465.6)	574.0 (1201.2)
Ozark, Ouachita-Appalachian forests	17,830	17.3 (32.5)	213.6 (352.8)	505.5 (1329.1)	197.2 (537.2)
Great plains	24,901	9.3 (95.9)	881.6 (1975.9)	309.2 (3307.9)	262.2 (793.2)
Mixed wood plains	14,228	57.6 (348.0)	87.1 (128.3)	430.0 (2109.3)	265.2 (679.5)
Northern forest	6035	77.0 (425.1)	56.5 (92.1)	752.1 (1431.1)	599.9 (1530.6)
Northwest forested mountains	209,173	72.2 (258.5)	119.9 (213.4)	417.6 (1078.5)	297.9 (546.6)
Mediterranean California	76,508	25.9 (110.3)	80.5 (164.7)	100.6 (252.2)	548.1 (847.9)
Marine west coast forest	3858	15.1 (20.8)	222.3 (262.8)	259.9 (412.8)	497.4 (543.2)

Values represent data from May to September.

We did not use road or parking data for three units (Channel Islands, Isle Royale, and Apostle Islands) because these parks are islands that do not have publicly accessible roads or parking.

### Microclimates within parks

**Figure 2:**
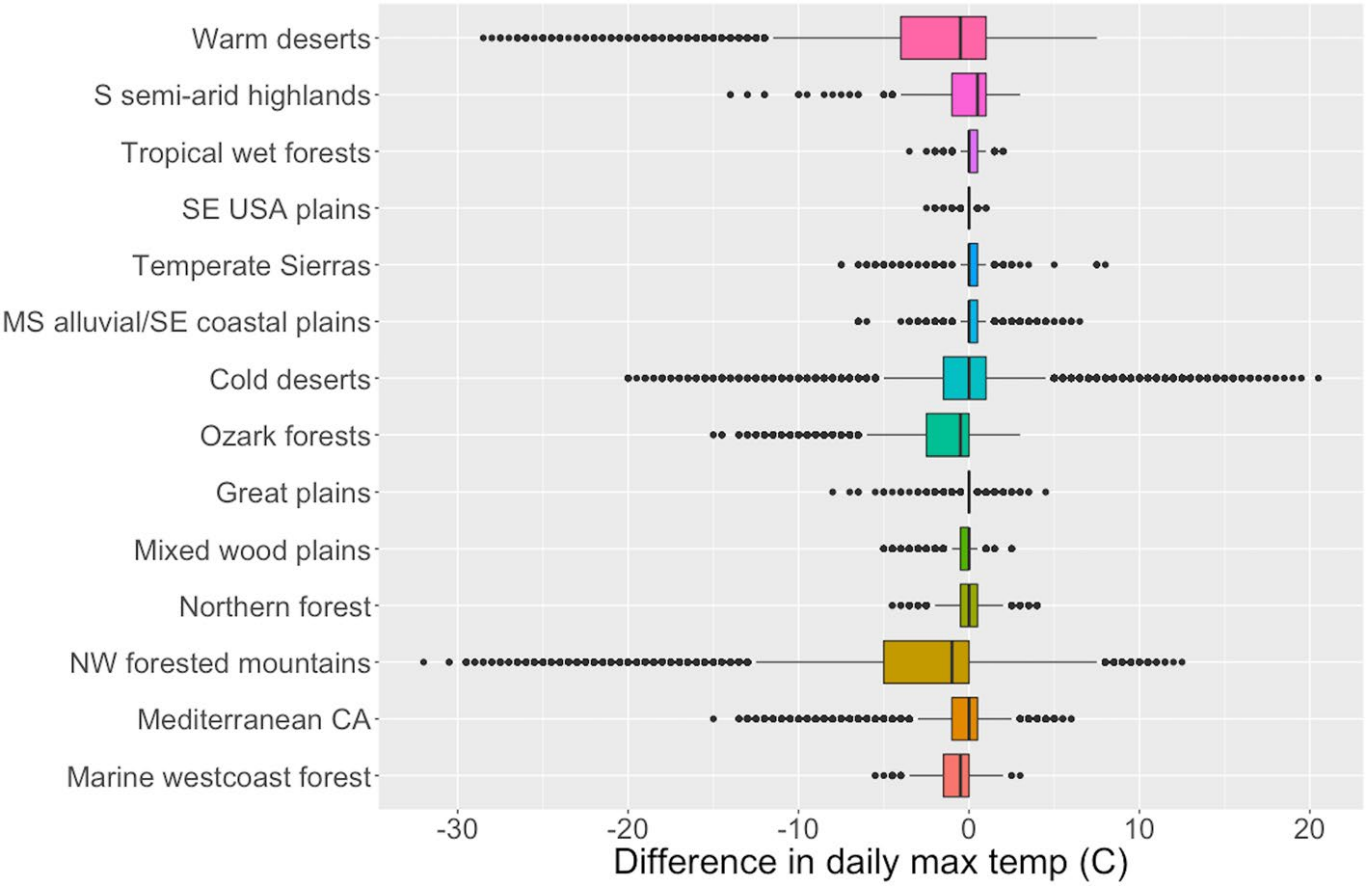
Boxplots of the distributions by ecoregion for the difference in daily maximum temperature (°C) between visitor centers and individual Flickr points within each park. Boxes represent the interquartile range, with black lines representing the medians; black dots represent outliers. Negative values indicate visitors are going to places within the park that are colder than the temperature at the visitor center.

Figure 2 shows the distributions for the difference in daily maximum temperature between the visitor center and individual Flickr point locations. Wider distributions (e.g., Northwest forested mountains ecoregion) indicate more microclimates within the parks, while narrower distributions (e.g., Southeastern USA plains) indicate daily temperatures are similar across the whole park unit. These microclimates represent the differences in temperature between where people visit compared to the visitor center; they do not necessarily represent differences in daily temperature across all park areas. Since some places may be inaccessible, we only explored temperature differences, and thus microclimates, in park areas that receive visitation.

Overall, there is less variation in the difference in daily precipitation between the visitor centers and Flickr point locations. At least 50% of the Flickr points had the same daily precipitation as the visitor centers in every ecoregion. However, there are still some differences in precipitation between Flickr points and visitor centers, with the Mississippi alluvial/Southeastern coastal plains ecoregion having the largest differences.

### Differences in visitation patterns between hot and cold days

**Figure 3:**
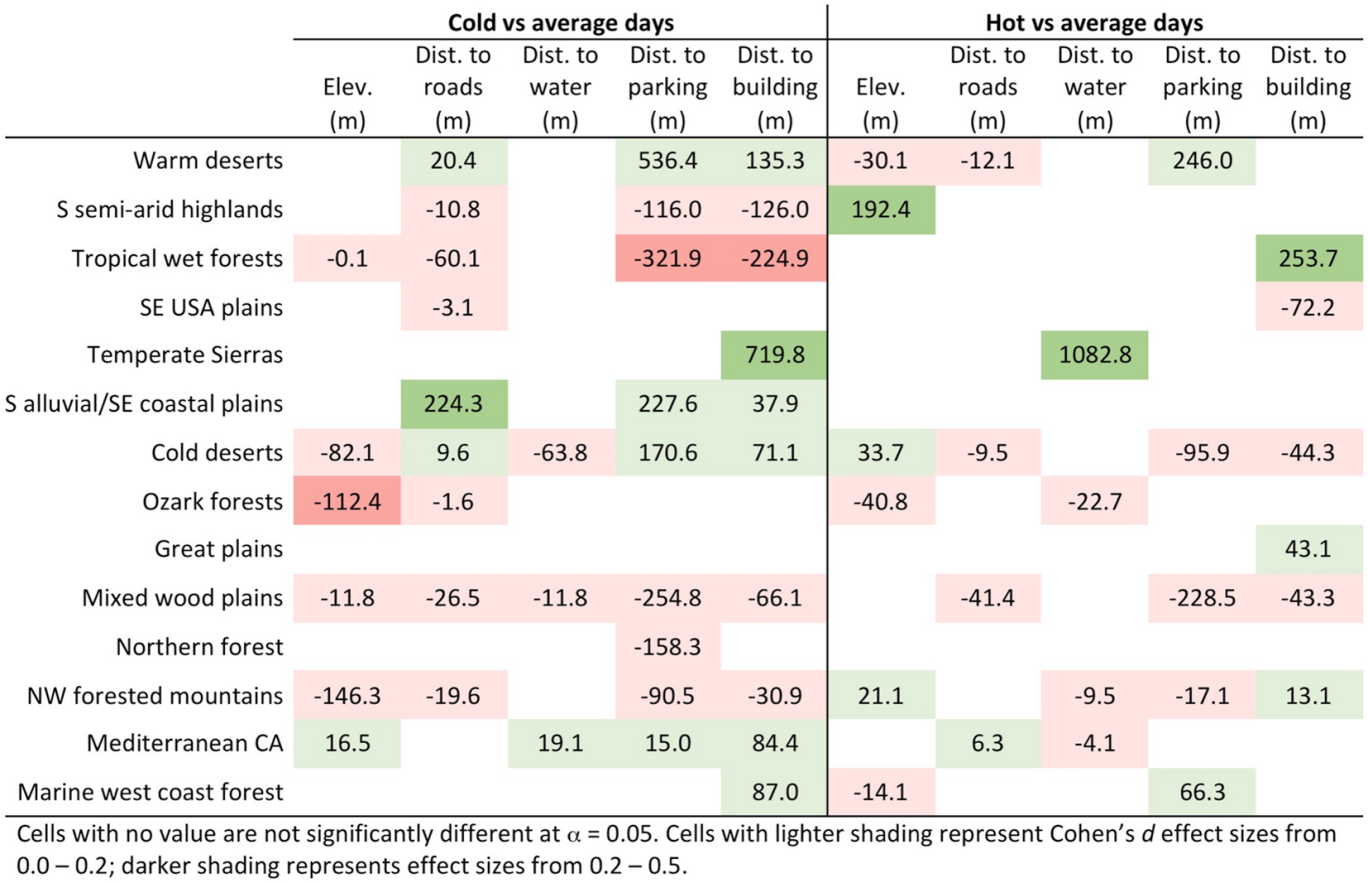
Differences in means on cold days, compared to average days (left side), and differences in means on hot days, compared to average days (right side). Positive values represent higher elevations and farther distance from features on cold or hot days (compared to average); negative values represent lower elevations and closer distance to features on hot or cold days.

The cutoff points for what was defined as a cold day, average day, and hot day differ by park unit and can be found in Supplementary Table A1. The effect of maximum temperature on visitors’ elevation and distance to roads, waterbodies, parking areas, and buildings varied by ecoregion (Fig. 3). There is not a consistent trend in how temperature impacts the spatial patterns of visitation across ecoregions for any variable. In some ecoregions (e.g., tropical wet forests, mixed wood plains), visitors stay closer to parking areas and buildings on cold days, but in other regions (e.g., cold deserts, warm deserts), visitors travel farther from infrastructure on cold days. Visitors tend to frequent lower elevations on cold days in most ecoregions, but there is not a consistent trend in elevation on hot days. Although temperature does affect visitors’ spatial distributions within parks, the effect sizes were all very small or small.

Boxes without values in Fig. 3 indicate there was no statistical differences across the three temperature classifications for that particular ecoregion; this does not necessarily mean no difference exists. Some ecoregions had smaller sample sizes (e.g., temperate Sierras at *n* = 797), while some had very large sample sizes (e.g., Northwest forested mountains at *n* = 209,173). Statistical power is higher when sample sizes are larger, so we were inherently more likely to detect significant differences in ecoregions with larger sample sizes. Sample sizes for each ecoregion based on temperature and precipitation grouping are available in Supplementary Table B1, and additional statistical information associated with Fig. 3 is available in Supplementary Table C1.

**Figure 4:**
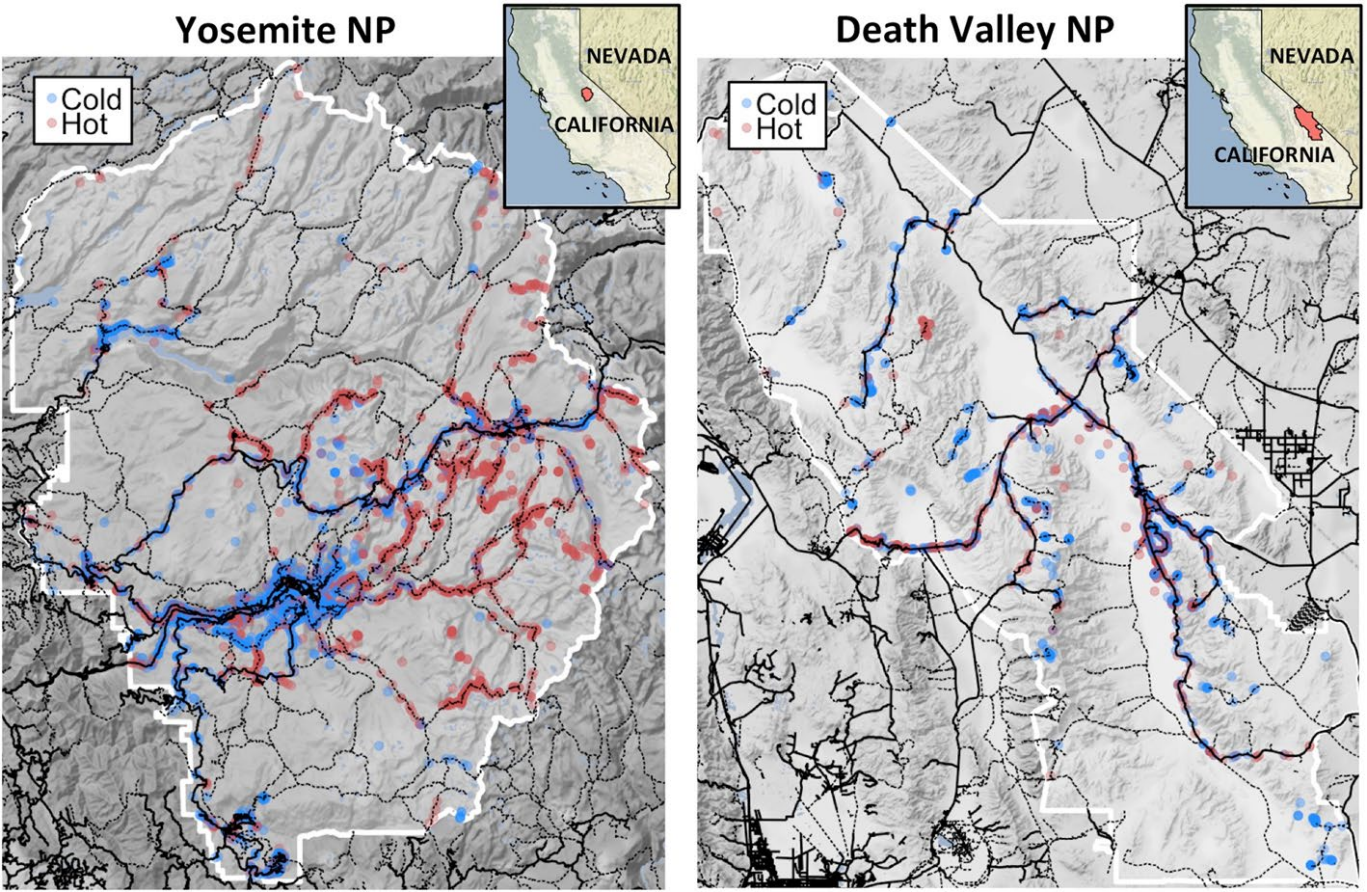
Spatial distribution of visitors in Yosemite National Park and Death Valley National Park on cold days (blue dots) compared to hot days (red dots). Solid black lines represent roads, and dotted black lines represent trails downloaded from OpenStreetMap. Figures created in R version 3.6.1 (www.r-project.org) with the ggmap package^42^.

Figure 4 shows examples of how spatial distributions differ during cold and hot days for two parks: Yosemite National Park (Northwest forested mountains ecoregion) and Death Valley National Park (warm deserts ecoregion). These maps suggest some trails or regions are more popular on hot days, while others are more popular on cold days. In Yosemite, the map shows visitors are more likely to stay closer to roads on cold days. This is consistent with findings from the results in Fig. 3 from the Northwest forested mountains ecoregion, that visitors stay 19.6 m closer to roads on cold days compared to average days. In Death Valley, visitors appear more likely to stay near roads on hot days, consistent with results from the warm deserts ecoregion that shows visitors stay 12.1 m closer to roads on hot days, and 20.4 m farther from roads on cold days, compared to average days. Maps showing general spatial distributions of visitors in each study site, as well as spatial distributions on cold versus hot days, are available online^41^.

### Differences in visitation patterns between wet and dry days

**Figure 5:**
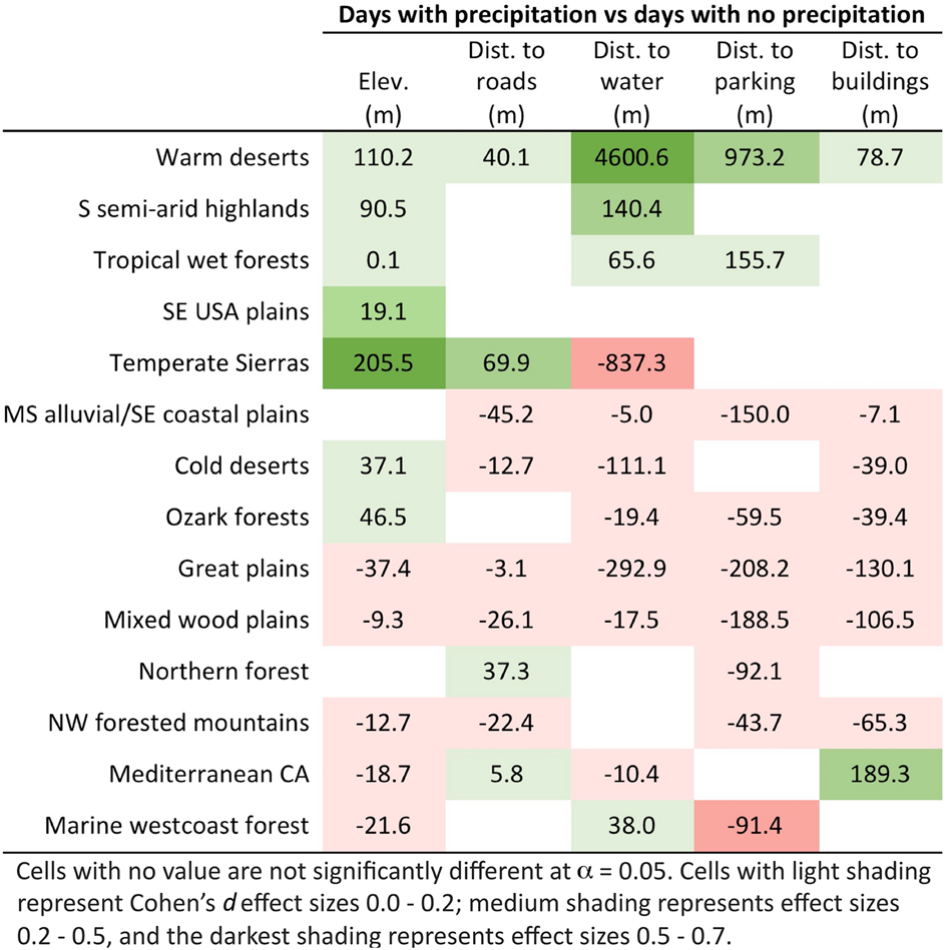
Differences in means on days with precipitation, compared to days with no precipitation. Positive values represent higher elevations and farther distance from features on days with precipitation; negative values represent lower elevations and closer distance to features on days with precipitation.

The effect of daily precipitation on visitors’ elevation and distance to roads, waterbodies, parking areas, and buildings also varied by ecoregion, although there are some trends across ecoregions (Fig. 5). Overall, on rainy days, visitors were more likely to stay near roads, waterbodies, parking areas, and buildings. However, this trend does not hold for some of the warmest ecoregions (e.g., warm deserts), where visitors were farther from infrastructure on rainy days. In the warmer ecoregions, visitors went to higher elevations on rainy days, but in the cooler ecoregions, visitors stayed at lower elevations on rainy days. Although rain does impact visitors’ spatial behavior in all ecoregions, the effect sizes are mostly very small, with a few effects being small or medium. Additional statistical information associated with Fig. 5 is available in Supplementary Table D1.

## Discussion

Our results suggest visitors do change where they go within NPS units based on daily temperature and precipitation. The effect of temperature on elevation and distance to a road, distance to a waterbody, distance to a parking area, and distance to a building varied by ecoregion, with no consistent trends across all ecoregions. Overall, visitors were more likely to stay near infrastructure and waterbodies on days with precipitation, although this is not true in every ecoregion. However, the effect sizes of the differences are mostly very small, indicating that maybe only a subset of visitors are impacted by weather. Weather impacts visitors differently depending on their activity type and demographic characteristics, so some visitors may be more or less impacted by the weather^43^. The majority of visitors stay very close to roads (i.e., over half are within 11 m from a road); it is possible weather may have less of an impact on visitors who plan to stay near roads, most likely very close to (if not in) a vehicle. More research is needed to determine if and why only certain groups of visitors alter their spatial behavior within parks based on the weather.

Climate change is expected to alter the total number of visitors to parks, with the majority of parks in the U.S. expected to see an increase in visitation^4^. This could strain park resources and cause overcrowding in some parks. Since most visitors stay close to roads, it is important to maintain the roads and infrastructure that are already present. Accommodating visitation demand may not require substantial increases in some types of outdoor recreation infrastructure (e.g., trails), but rather a re-thinking of what the typical park experience is for most visitors. With most visitors choosing to stay extremely close to existing park infrastructure, capital investments should be focused on infrastructure upgrades and developments (e.g., remodeling and expanding visitor centers) that are better able to serve the needs and desires of more visitors in the future.

Previous work has found total visitation to parks is influenced by daily and monthly weather conditions^6,9^. Our findings suggest some visitors will respond to warmer than average temperatures by adapting where they go within a park. For example, some visitors may go to higher elevations on warm days, while other parks may see more visitors at lower elevations, possibly in cooler canyons or near the ocean. In some ecoregions, visitors may also choose to stay closer to roads or bodies of water on exceptionally hot days. Once a visitor is already at a park unit, they can respond to adverse weather by not visiting (i.e., staying in nearby towns), visiting a different location in the park, or changing activities^43^. More research is needed to understand how visitors decide to respond in different ways, and how that varies by user group. Park managers can help visitors adapt to extreme temperatures by providing information on which areas of the park, that are accessible by road, are comparatively cooler. However, not all parks contain microclimates that may allow for adaptation.

Parks in some ecoregions have more microclimates than others. Our analyses showed parks in the warm deserts, cold deserts, and the Northwest forested mountains ecoregions had wide distributions in the difference in temperature between visitors’ locations in the park and the temperature at the visitor center. In other ecoregions, such as the Southeast USA plains, visitors were almost always at a location in the park that had the same temperature as the visitor center. Visitors may therefore have a greater ability to adapt and spatially substitute outdoor recreation settings within park boundaries at some parks compared to others. However, we only investigated microclimates with regards to where people currently visit; it is possible that some parks in this study do have microclimates within their boundaries that are not currently visited, but may see visitation in the future. In parks that do not have varying microclimates, visitors may be less likely to visit on days with unfavorable temperatures rather than change their spatial behavior within the park. This is consistent with previous research showing visitation declined in some Utah national parks once temperatures were above 25 °C, but visitation continued to increase above this threshold in parks that seemingly had more microclimates^6^.

Although this analysis only covered the summer season, it is likely that some trends may be attributed to within-season variability. For instance, it is more likely to be cold in May and September, and hot in July and August. In some mountainous parks, certain roads or trails may be closed at the beginning of the summer season until snow melts. Therefore, visitors may not have had the option to visit some park areas on colder than average days. Parks in the Northwest forested mountains ecoregion are the most likely to have areas closed due to snow, so these managerial factors are likely to have the biggest influence in this ecoregion. In some parks, visitors’ spatial behavior may be driven by managerial factors (i.e., closed roads or trails) rather than solely visitors’ decisions.

As with any data source, social media has its limitations. Social media may not be representative of the spatial patterns of all park visitors, since only a small portion of total visitors post photos to Flickr^32,44^. Additionally, some parks tend to have substantially more social media posts than other parks, indicating the most popular parks were overrepresented in this analysis. We explored the impact of weather on visitors at the ecoregion level; however, future research is needed to determine if there is additional variation across parks within the same ecoregion. OpenStreetMap was an excellent resource for large-scale volunteered geographic information, but the accuracy of this data source does vary by location and feature^45–47^. While the road and water features appeared to be complete across all NPS units in this study, the parking and building datasets were likely not entirely complete. In other words, some buildings and parking areas were missing, but all of the parking areas and buildings documented on OpenStreetMap did exist in that location. Therefore, the estimates for distances to parking and buildings likely represent high estimates. In addition, distances to features do not necessarily indicate how far a visitor hikes or ventures; a visitor could hike for over 500 m and still be within 10 m of a road.

Our investigation began with an effort to understand how weather may impact visitors’ spatial behavior across NPS units. Further studies could explore if weather changes spatial patterns of visitors outside park boundaries, such as to gateway towns and surrounding parklands. Additionally, future work could explore how weather impacts spatial patterns of visitors to parks in other countries. This approach of using social media data to understand spatial patterns could be replicated in other locations that have daily weather data. We found that the effect of daily weather on visitation patterns was not homogenous across the U.S. Our results indicated large differences across ecoregions, so results from one ecoregion cannot necessarily be extrapolated onto parks with differing climates or topography. We would expect parks in other countries may exhibit comparable results to the ecoregion that has the most similar climate and topography; however, this needs additional research. In addition, this analysis demonstrates the utility of social media for revealing visitation patterns within parks at high spatial and temporal resolutions, which can be useful to understand visitor behavior beyond the context of weather-dependencies^13^.

## Conclusions

In certain ecoregions, visitors alter the locations they go to within NPS units based on daily weather conditions. The effect of temperature and precipitation on visitors’ spatial behavior varies by ecoregion, likely because the climates, topography, and availability of microclimates within parks differ by these ecoregions. Some parks may see an increase in visitors to higher elevations on hot days, while other parks may see more visitors at lower elevations on hot days. Visitors are overall more likely to stay near infrastructure on rainy days. Park managers should expect spatial distributions of summer visitors within parks to change in the future due to increasing numbers of hot days. In parks that contain more microclimates, visitors may have a greater ability to adapt to adverse temperature conditions by spatially substituting one outdoor recreation setting for another.

## Methods

### Study sites

Study sites include all NPS units in the continental U.S. larger than 10,000 acres (4047 hectares). NPS units include national parks, national monuments, national recreation areas, and national seashores, among others. Each park unit was assigned both a level I and a level II ecoregion based on the location of the centroid of the unit. Level I ecoregions represent the most general category, while level II ecoregions are more detailed. For nearly all ecoregions we used the level I ecoregions. However, two level I ecoregions (North American deserts and Eastern temperate forests) were split into their level II ecoregions due to their vast size and the number of study sites contained within them. Figure 1 shows the study sites along with the ecoregion categories used in this paper; a full list of all NPS units included in this study and their ecoregion classifications can be found in the Supplementary Table E1.

### Data collection and processing

All data used in this paper are publicly available. Table 3 lists all datasets used along with their sources. In cases where an R package is listed as a source, we downloaded the data directly through R, using the specified packages to interact with the Application Programming Interfaces (APIs). All R code written for data collection, processing, and analysis is available^41^.

**Table 3:** Datasets and sources used in this paper.

Data	Type of data	Source	Citation
NPS spatial boundaries	Polygons	NPS	^48^
NPS unit centroids	Table (turned into points from lat/long)	NPS	^49^
Main visitor center for each NPS unit	Table (turned into points from lat/long)	Manually compiled via Google Maps and NPS unit websites	Dataset made available at^41^
Acreage of NPS units	Table	NPS	^50^
Visitation at NPS units	Table	NPS	^51^
Ecoregions levels I and II	Polygons	EPA	^11^
Geotagged Flickr posts (2006–2018)	Points	Flickr API (via Python code)	^52^
Daily temperature and precipitation (2006–2018)	Raster (1 km resolution)	Daymet R package: daymetr	^53^ R package:^54^
Elevation	Raster (1/3 arcsec resolution)	USGS R package: elevatr	^55^ R package:^56^
Roads	Lines^a^	OpenStreetMap R package: osmdata	^57^ R package:^58^
Parking areas	Polygons and multipolygons		
Bodies of water	Polygons, multipolygons, and lines		
Buildings	Polygons and multipolygons		

^a^These data also include raw polygon files (representing loop roads) that were converted to line features.

We downloaded Flickr data within the study sites between May and September, from 2006 to 2018, from the Flickr API using Python. We downloaded these data in October 2019. We deleted any photos by the same user, on the same day, within 10 m of another photo posted by the same user; therefore, we only retained one photo per user, per location. This is similar to the concept of PUDs^29,30^, except we only deleted duplicates in close proximity rather than duplicates anywhere within the unit. We did this believing it was important to retain posts by the same user if they were in different locations within the park. Sample sizes by unit are available in Supplementary Table F1.

We joined each Flickr point to the daily weather on that day at that location using weather data from Daymet. Daymet contains weather data for every location in the continental U.S., which is modeled from individual weather station data, and has a high accuracy^53,59^. Our analysis does not include any Flickr points tagged in an ocean (e.g., off the coast of a national park) because Daymet does not provide weather estimates over oceans. We also connected each Flickr point to the elevation at that particular location. We downloaded data on roads, waterbodies, parking areas, and buildings from OpenStreetMap in December 2019 (specific information on download criteria is in Supplementary Table G1). For each Flickr point, we calculated the straight-line distance to the nearest road, waterbody, parking area, and building.

### Analysis

#### Social media data validation

We compared the number of Flickr PUDs within each unit between the months of May and September from 2006 to 2018 to the NPS-reported visitation for each unit during the same time period to ensure the Flickr data are a reliable and representative indicator of visitation. PUD indicates that only one photo per visitor was counted each day; duplicate posts by the same visitor on the same day were removed even if they were in different areas of the park. Subsequent analyses used the full dataset filtered to include just one photo per user, per location. We obtained Spearman’s correlation coefficient as a measure of association between Flickr PUD and NPS-reported visitation. We used Spearman’s rank correlation because the distributions were found to be non-normal after running a Shapiro–Wilk test. Two parks were not included in this analysis because NPS did not have visitation data for these parks during this time period.

#### Understanding how weather impacts visitors’ spatial behavior

We first explored if and how individual parks have different microclimates (i.e., the park offers different areas where visitors can go that may have slightly different climates). We recorded the differences between the daily maximum temperature and precipitation at Flickr points compared to the main visitor center on that day. We plotted distributions of differences by ecoregion to see if visitors were going to places within parks that have substantially different weather than at the visitor centers.

We then investigated the effect of maximum temperature and precipitation on visitors’ spatial behavior by grouping visitors by the weather during the day they visited. For maximum temperature, visitors were grouped into three categories: cold day, average day, or hot day, based on the temperature at the visitor center on the day of the visit. Average days were defined as those within one standard deviation from the unit-specific seasonal mean maximum temperature. Cold days were defined as days with a maximum temperature lower than one standard deviation below the unit-specific seasonal mean maximum temperature. Hot days were classified as days with a maximum temperature greater than one standard deviation above the unit-specific seasonal mean maximum temperature. We grouped these observations by unit rather than ecoregion to reduce bias. For instance, one park within an ecoregion could be warmer than the others; grouping by unit avoids having all data from one park classified in the same temperature category. Precipitation was split into two groups based on whether or not there was precipitation at the visitor center on the day of the visit.

We tested if maximum temperature or precipitation affected: (1) the elevations visitors were traveling to within a park; (2) their distance to roads; (3) their distance to waterbodies; and (4) their distance to designated parking areas or buildings. We ran Welch’s ANOVA tests to determine if there were differences in the spatial patterns between cold, average, and hot groups. If the results were significant at the 0.05 level, we ran Games-Howell post-hoc tests to determine where the significant differences were (i.e., if differences were between the cold and average group, hot and average, hot and cold, or all three). We used Games–Howell tests because they do not require the assumptions of equal variances or equal sample sizes to be met^60^. Additionally, if there were significant differences between groups, we reported Cohen’s *d* to measure how large the effect size was. For precipitation, we ran Welch’s t-tests with Cohen’s *d* effect sizes. Welch’s tests were used rather than Student’s t-tests and standard ANOVAs because much of the data violated the assumption of equal variances^61^. We ran separate tests for each ecoregion, given weather may impact visitors differently by ecoregion. To visually compare how distributions may differ, we mapped spatial distributions in parks on cold days compared to hot days.

## Supplementary Information

Supplementary Information.

## Data Availability

The individual datasets are all publicly available, as described in Table 3. The final compiled dataset used in this paper, and the code written for this analysis, can be found at: https://doi.org/10.3886/E119191V1.

## References

[1] Hand, M. S., Smith, J. W., Peterson, D. L., Brunswick, N. A. & Brown, C. P. Effects of climate change on outdoor recreation. In *Climate Change Vulnerability and Adaptation in the Intermountain Region: Part 2* (eds. Halofsky, J. E., Peterson, D. L., Ho, J. J., Little, N., J., & Joyce, L. A.). *Gen. Tech. Rep. RMRS-GTR-375.* **375**, 316–338 (US Department of Agriculture, 2018).

[2] Hammer T, Mose I, Siegrist D, Weixlbaumer N (2016). Parks of the Future: Protected Areas in Europe Challenging Regional and Global Change.

[3] Hewer MJ, Gough WA (2018). Thirty years of assessing the impacts of climate change on outdoor recreation and tourism in Canada. Tour. Manag. Perspect..

[4] Fisichelli NA, Schuurman GW, Monahan WB, Ziesler PS (2015). Protected area tourism in a changing climate: Will visitation at US national parks warm up or overheat?. PLoS ONE.

[5] Millhäusler A, Anderwald P, Haeni M, Haller RM (2016). Publicity, economics and weather—Changes in visitor numbers to a European National Park over 8 years. J. Outdoor Recreat. Tour..

[6] Smith JW, Wilkins E, Gayle R, Lamborn CC (2018). Climate and visitation to Utah's ‘Mighty 5’national parks. Tour. Geograph..

[7] Manning, R. E. *Studies in Outdoor Recreation: Search and Research for Satisfaction*. (Oregon State University Press, Oregon, 2010).

[8] National Park Service. *NPS Public Use Statistics Query Builder* https://irma.nps.gov/STATS/SSRSReports/NationalReports/Query Builder for Public Use Statistics (1979—Last Calendar Year) (2020).

[9] Paudyal R, Stein TV, Birendra K, Adams DC (2019). Effects of weather factors on recreation participation in a humid subtropical region. Int. J. Biometeorol..

[10] National Park Service. *About Us: National Park System* https://www.nps.gov/aboutus/national-park-system.htm (2020).

[11] U.S. Environmental Protection Agency. *Ecoregions of North America* https://www.epa.gov/eco-research/ecoregions-north-america (2016).

[12] Tennekes M (2018). tmap: Thematic maps in R. J. Stat. Softw..

[13] da Mota VT, Pickering C (2020). Using social media to assess nature-based tourism: Current research and future trends. J. Outdoor Recreat. Tour..

[14] Hewer MJ, Scott D, Fenech A (2016). Seasonal weather sensitivity, temperature thresholds, and climate change impacts for park visitation. Tour. Geograph..

[15] Scott D, Lemieux C (2010). Weather and climate information for tourism. Proc. Environ. Sci..

[16] Becken S, Wilson J (2013). The impacts of weather on tourist travel. Tour. Geograph..

[17] Scott D, Gössling S, de Freitas CR (2008). Preferred climates for tourism: Case studies from Canada, New Zealand and Sweden. Clim. Res..

[18] Steiger R, Abegg B, Jänicke L (2016). Rain, rain, go away, come again another day. Weather preferences of summer tourists in mountain environments. Atmosphere.

[19] Rutty M, Scott D (2010). Will the Mediterranean become “too hot" for tourism? A reassessment. Tour. Hosp. Plan. Dev..

[20] Dubois G, Ceron J-P, Gössling S, Hall CM (2016). Weather preferences of French tourists: Lessons for climate change impact assessment. Clim. Change.

[21] Hewer MJ, Scott D, Gough WA (2015). Tourism climatology for camping: A case study of two Ontario parks (Canada). Theoret. Appl. Climatol..

[22] Gössling S, Scott D, Hall CM, Ceron J-P, Dubois G (2012). Consumer behaviour and demand response of tourists to climate change. Ann. Tour. Res..

[23] Hadwen WL, Arthington AH, Boon PI, Taylor B, Fellows CS (2011). Do climatic or institutional factors drive seasonal patterns of tourism visitation to protected areas across diverse climate zones in eastern Australia?. Tour. Geograph..

[24] Monahan WB (2016). Climate change is advancing spring onset across the U.S. national park system. Ecosphere..

[25] Rutty M, Scott D (2014). Thermal range of coastal tourism resort microclimates. Tour. Geograph..

[26] Ghermandi A, Sinclair M (2019). Passive crowdsourcing of social media in environmental research: A systematic map. Glob. Environ. Change.

[27] Wilkins EJ, Wood SA, Smith JW (2020). Uses and limitations of social media to inform visitor use management in parks and protected areas: A systematic review. Environ. Manag..

[28] Keeler BL (2015). Recreational demand for clean water: Evidence from geotagged photographs by visitors to lakes. Front. Ecol. Environ..

[29] Sessions C, Wood SA, Rabotyagov S, Fisher DM (2016). Measuring recreational visitation at U.S. National Parks with crowd-sourced photographs. J. Environ. Manag..

[30] Wood SA, Guerry AD, Silver JM, Lacayo M (2013). Using social media to quantify nature-based tourism and recreation. Sci. Rep..

[31] Leggett C, Horsch E, Smith C, Unsworth R (2017). Estimating Recreational Visitation to Federally-Managed Lands.

[32] Barros C, Moya-Gómez B, Gutiérrez J (2019). Using geotagged photographs and GPS tracks from social networks to analyse visitor behaviour in national parks. Curr. Issues Tour..

[33] Sinclair M, Mayer M, Woltering M, Ghermandi A (2020). Using social media to estimate visitor provenance and patterns of recreation in Germany's national parks. J. Environ. Manage..

[34] Toivonen T (2019). Social media data for conservation science: A methodological overview. Biol. Cons..

[35] National Coordination Office for Space-Based Positioning Navigation and Timing. *GPS Accuracy* https://www.gps.gov/systems/gps/performance/accuracy/ (2017).

[36] Walden-Schreiner C, Leung Y-F, Tateosian L (2018). Digital footprints: Incorporating crowdsourced geographic information for protected area management. Appl. Geogr..

[37] Hale BW (2018). Mapping potential environmental impacts from tourists using data from social media: A case study in the Westfjords of Iceland. Environ. Manag..

[38] Schirpke U, Meisch C, Marsoner T, Tappeiner U (2018). Revealing spatial and temporal patterns of outdoor recreation in the European Alps and their surroundings. Ecosyst. Serv..

[39] Walden-Schreiner C, Rossi SD, Barros A, Pickering C, Leung Y-F (2018). Using crowd-sourced photos to assess seasonal patterns of visitor use in mountain-protected areas. Ambio.

[40] Donahue ML (2018). Using social media to understand drivers of urban park visitation in the Twin Cities, MN. Landsc. Urban Plan..

[41] Wilkins, E. J. & Smith, J. W. Weather & summer spatial behavior of U.S. national park visitors (Flickr data 2006–2018). *Inter-university Consortium for Political and Social Research*. https://doi.org/10.3886/E119191V1 (2020).

[42] Kahle D, Wickham H (2013). ggmap: Spatial visualization with ggplot2. R J..

[43] Verbos RI, Altschuler B, Brownlee MT (2018). Weather studies in outdoor recreation and nature-based tourism: A research synthesis and gap analysis. Leisure Sci..

[44] Muñoz L, Hausner VH, Runge C, Brown G, Daigle R (2020). Using crowdsourced spatial data from Flickr vs. PPGIS for understanding nature's contribution to people in Southern Norway. People Nat..

[45] Zhang, H. & Malczewski, J. Accuracy evaluation of the Canadian OpenStreetMap road networks. *Int. J. Geospat. Environ. Res.* **5** (2017).

[46] Parr, D. A. *The production of volunteered geographic information: A study of OpenStreetMap in the United States* Ph.D. thesis, Texas State University (2015).

[47] Haklay M (2010). How good is volunteered geographical information? A comparative study of OpenStreetMap and Ordnance Survey datasets. Environ. Plan. B Plan. Des..

[48] National Park Service. *Administrative boundaries of National Park System Units 9/30/2019*. https://irma.nps.gov/DataStore/Reference/Profile/2224545?lnv=True (2019).

[49] National Park Service. *Park Unit Centroids* https://public-nps.opendata.arcgis.com/datasets/nps-boundary-centroids-1 (2017).

[50] National Park Service. *National Park Service Acreage Reports: Calendar Year 2018*. https://www.nps.gov/subjects/lwcf/acreagereports.htm (2019).

[51] National Park Service. *Annual visitation report by years: 2008 to 2018*. https://irma.nps.gov/Stats/SSRSReports/National%20Reports/Annual%20Visitation%20By%20Park%20(1979%20-%20Last%20Calendar%20Year) (2019).

[52] flickr. *The Flickr developer guide: API* https://www.flickr.com/services/developer/api/ (n.d.).

[53] Thornton, P. E. *et al.* Daymet: Daily surface weather data on a 1-km grid for North America, Version 3. https://doi.org/10.3334/ORNLDAAC/1328 (2016).

[54] Hufkens K (2018). An integrated phenology modelling framework in R: Modelling vegetation phenology with phenor. Methods Ecol. Evol..

[55] U.S. Geological Survey. *The National Map—elevation point query service* https://ned.usgs.gov/epqs/ (2017).

[56] Hollister, J. W. & Shah, T. *elevatr: Access elevation data from various APIs* https://rdrr.io/cran/elevatr/ (2018).

[57] OpenStreetMap Contributors. *Planet OSM* https://www.openstreetmap.org (2019).

[58] Padgham, M., Lovelace, R., Salmon, M. & Rudis, B. osmdata. *J. Open Source Softw.* **2** (2017).

[59] Behnke R (2016). Evaluation of downscaled, gridded climate data for the conterminous United States. Ecol. Appl..

[60] Hilton A, Armstrong RA (2006). Statnote 6: Post-hoc ANOVA tests. Microbiologist.

[61] Delacre, M., Lakens, D. & Leys, C. Why psychologists should by default use Welch’s t-test instead of Student’s t-test. *Int. Rev. Soc. Psychol.* **30** (2017).

