# Supplementary material for: Social media reveal ecoregional variation in how weather influences visitor behavior in U.S. National Park Service units
Source: Sci Rep. 2021 Jan 28;11:2403. doi: 10.1038/s41598-021-82145-z (PMC7843642; doi:10.1038/s41598-021-82145-z)
Supplement: Supplementary file 1 — Supplementary Information. [file 41598_2021_82145_MOESM1_ESM.docx]

**Supplementary material for the paper:** Wilkins, E. J., Howe, P. D., & Smith, J. W. Social media reveal ecoregional variation in how weather influences visitor behavior in U.S. National Park Service units.

**List of Appendices**

**APPENDIX A.** Temperature categorization by park.

**APPENDIX B.** Sample sizes based on temperature and precipitation groupings.

**APPENDIX C.** Full statistical results associated with Figure 3.

**APPENDIX D.** Full statistical results associated with Figure 5.

**APPENDIX E.** Study sites by ecoregion.

**APPENDIX F.** Sample sizes for each study site.

**APPENDIX G.** Key-value pairs used to download OpenStreetMap data.

**APPENDIX A.** Temperature categorization by park.

**Supplementary Table A1** Maximum daily temperature ranges for what is considered a cold, average, or hot day, by park unit. Average days are within one standard deviation of the mean, while cold days are greater than one standard deviation colder, and hot days are greater than one standard deviation warmer.

| **Unit** | **Ecoregion** | **Cold**  **range (ºC)** | **Average range (ºC)** | **Hot**  **range (ºC)** |
| --- | --- | --- | --- | --- |
| ACAD | Mixed wood plains | 9 - 19 | 19.5 - 27 | 27.5 - 33.5 |
| CUVA | Mixed wood plains | 11 - 21.5 | 22 - 30 | 30.5 - 36 |
| INDU | Mixed wood plains | 10.5 - 20.5 | 21 - 29.5 | 30 - 38 |
| SLBE | Mixed wood plains | 9 - 20 | 20.5 - 28.5 | 29 - 33.5 |
| AMIS | Warm deserts | 27.5 - 31 | 31.5 - 37.5 | 39 - 41 |
| BIBE | Warm deserts | 16 - 28.5 | 29 - 36 | 36.5 - 40.5 |
| DEVA | Warm deserts | 25.5 - 36.5 | 37 - 47.5 | 48 - 50 |
| JOTR | Warm deserts | 18 - 26 | 26.5 - 37.5 | 38 - 43.5 |
| LAKE | Warm deserts | 20.5 - 32 | 32.5 - 42.5 | 43 - 48 |
| MOJA | Warm deserts | 21 - 30 | 30.5 - 41 | 41.5 - 45.5 |
| ORPI | Warm deserts | NA (no obs.) | 25 - 35.5 | 36 - 42 |
| RIGR | Warm deserts | 25 - 34 | 34.5 - 40.5 | 41 - 43 |
| WHSA | Warm deserts | 18.5 - 27.5 | 28 - 35 | 35.5 - 41 |
| APIS | Northern forest | 11.5 - 19.5 | 20 - 27.5 | 28 - 32 |
| ISRO | Northern forest | 9 - 19 | 19.5 - 26 | 26.5 - 30.5 |
| PIRO | Northern forest | 4.5 - 18.5 | 19 - 26.5 | 27 - 32.5 |
| SACN | Northern forest | 11 - 21.5 | 22 - 29.5 | 30 - 35 |
| UPDE | Northern forest | 11 - 21.5 | 22 - 29.5 | 30 - 35.5 |
| VOYA | Northern forest | 10.5 - 19 | 20 - 27.5 | 28 - 30.5 |
| ARCH | Cold deserts | 13.5 - 26 | 26.5 - 36.5 | 37 - 42.5 |
| BLCA | Cold deserts | 4.5 - 18 | 18.5 - 30 | 30.5 - 32.5 |
| BRCA | Cold deserts | 4.5 - 17.5 | 18 - 27.5 | 28 - 32.5 |
| CACH | Cold deserts | 16 - 26 | 26.5 - 33.5 | 34 - 37 |
| CANY | Cold deserts | 9.5 - 21 | 21.5 - 32.5 | 33 - 37.5 |
| CARE | Cold deserts | 11.5 - 23 | 23.5 - 33 | 33.5 - 37.5 |
| CHCU | Cold deserts | 13.5 - 24 | 25 - 32 | 32.5 - 36 |
| CIRO | Cold deserts | 8 - 18 | 19 - 29.5 | 30 - 33.5 |
| COLM | Cold deserts | 11.5 - 24 | 24.5 - 33 | 33.5 - 37.5 |
| CRMO | Cold deserts | 7 - 20 | 20.5 - 31.5 | 32 - 36 |
| DINO | Cold deserts | 13 - 23.5 | 24 - 34 | 34.5 - 37.5 |
| ELMA | Cold deserts | 12 - 23 | 24 - 32 | 32.5 - 34.5 |
| GLCA | Cold deserts | 14 - 27.5 | 28 - 37.5 | 38 - 43 |
| GRBA | Cold deserts | 10.5 - 26 | 26.5 - 34.5 | 35 - 38.5 |
| GRCA | Cold deserts | 5.5 - 19.5 | 20 - 29 | 29.5 - 35.5 |
| GRSA | Cold deserts | 9 - 20 | 20.5 - 28 | 28.5 - 32 |
| LARO | Cold deserts | 13.5 - 23 | 23.5 - 33.5 | 34 - 40.5 |
| MEVE | Cold deserts | 7 - 20.5 | 21 - 30.5 | 31 - 34 |
| PEFO | Cold deserts | 14 - 25 | 25.5 - 33.5 | 34 - 38.5 |
| WUPA | Cold deserts | 13 - 25.5 | 26 - 35.5 | 36 - 40.5 |
| ZION | Cold deserts | 14 - 26 | 26.5 - 37 | 37.5 - 42.5 |
| ASIS | MS alluvial/SE coastal plains | 11.5 - 23.5 | 24 - 31.5 | 32 - 37.5 |
| CACO | MS alluvial/SE coastal plains | 8.5 - 19.5 | 20 - 27 | 27.5 - 33.5 |
| CAHA | MS alluvial/SE coastal plains | 17 - 25.5 | 26 - 30.5 | 31 - 34 |
| CALO | MS alluvial/SE coastal plains | 21.5 - 27 | 27.5 - 31.5 | 32 - 36.5 |
| CANA | MS alluvial/SE coastal plains | 25.5 - 28.5 | 29 - 32.5 | 33 - 36 |
| CUIS | MS alluvial/SE coastal plains | 24.5 - 28 | 28.5 - 33 | 33.5 - 37.5 |
| FIIS | MS alluvial/SE coastal plains | 9.5 - 22.5 | 23 - 29.5 | 30 - 40 |
| GATE | MS alluvial/SE coastal plains | 11 - 22.5 | 23 - 31 | 31.5 - 39 |
| GUIS | MS alluvial/SE coastal plains | 22.5 - 29 | 29.5 - 33.5 | 34 - 38 |
| JELA | MS alluvial/SE coastal plains | 25 - 28.5 | 29 - 33.5 | 34 - 38 |
| TIMU | MS alluvial/SE coastal plains | 24 - 28.5 | 29 - 33.5 | 34 - 36.5 |
| BADL | Great plains | 7.5 - 23.5 | 24 - 34 | 34.5 - 42.5 |
| BICA | Great plains | 10.5 - 19 | 20 - 32.5 | 33 - 37.5 |
| LAMR | Great plains | 23.5 - 26.5 | 30.5 - 36 | 38 - 39 |
| MISS | Great plains | 5 - 20.5 | 21 - 30 | 30.5 - 37 |
| MNRR | Great plains | 15 - 24.5 | 25 - 32 | 32.5 - 38.5 |
| NIOB | Great plains | 17 - 27.5 | 28 - 35 | 36 - 37.5 |
| PAIS | Great plains | 25 - 29.5 | 30 - 33 | 33.5 - 35.5 |
| SAND | Great plains | 18 - 24 | 26 - 34 | NA (no obs.) |
| TAPR | Great plains | 18.5 - 25 | 25.5 - 34 | 34.5 - 39.5 |
| THRO | Great plains | 6.5 - 21.5 | 22 - 32 | 32.5 - 38.5 |
| BAND | NW forested mountains | 15.5 - 22 | 22.5 - 32 | 32.5 - 36 |
| CRLA | NW forested mountains | -2 - 13 | 13.5 - 23.5 | 24 - 29 |
| CURE | NW forested mountains | 9 - 21.5 | 22 - 29.5 | 30 - 33 |
| GLAC | NW forested mountains | 6.5 - 21 | 21.5 - 31 | 31.5 - 37.5 |
| GRTE | NW forested mountains | 2 - 18 | 18.5 - 28.5 | 29 - 34 |
| JODA | NW forested mountains | 13 - 24 | 24.5 - 36.5 | 37 - 40 |
| KICA | NW forested mountains | 1.5 - 19.5 | 20 - 29 | 29.5 - 32.5 |
| LABE | NW forested mountains | 9 - 21 | 21.5 - 32 | 33 - 36.5 |
| LACH | NW forested mountains | 16 - 21 | 21.5 - 29 | 29.5 - 36.5 |
| LAVO | NW forested mountains | 1 - 19 | 19.5 - 27 | 27.5 - 31.5 |
| MORA | NW forested mountains | 1 - 11.5 | 12 - 21.5 | 22 - 29 |
| NOCA | NW forested mountains | 11 - 19.5 | 20 - 30.5 | 31 - 37 |
| OLYM | NW forested mountains | 10.5 - 16.5 | 17 - 24 | 24.5 - 34 |
| ROLA | NW forested mountains | 11.5 - 18 | 18.5 - 29 | 29.5 - 38 |
| ROMO | NW forested mountains | 1 - 19 | 19.5 - 27.5 | 28 - 32 |
| SEQU | NW forested mountains | 14.5 - 29.5 | 30 - 38.5 | 39 - 46 |
| WHIS | NW forested mountains | 15.5 - 24.5 | 25.5 - 38 | 38.5 - 41 |
| WICA | NW forested mountains | 9.5 - 21.5 | 22 - 32.5 | 33 - 39.5 |
| YELL | NW forested mountains | 2 - 15 | 15.5 - 25.5 | 26 - 31.5 |
| YOSE | NW forested mountains | 7.5 - 23.5 | 24 - 34 | 34.5 - 39.5 |
| BICY | Tropical wet forests | 27.5 - 31.5 | 32 - 34.5 | 35 - 36.5 |
| BISC | Tropical wet forests | 27.5 - 28.5 | 29 - 32.5 | 33 - 33.5 |
| EVER | Tropical wet forests | 27.5 - 30.5 | 31 - 33.5 | 34 - 35 |
| BISO | Ozark forests | 10.5 - 19.5 | 20 - 29.5 | 30 - 33 |
| BUFF | Ozark forests | 6.5 - 24.5 | 25 - 34 | 34.5 - 39.5 |
| CUGA | Ozark forests | 18 - 23.5 | 24 - 32 | 32.5 - 36 |
| DEWA | Ozark forests | 11 - 21 | 21.5 - 29 | 29.5 - 35 |
| GARI | Ozark forests | 21 - 22 | 24.5 - 28 | 30.5 - 32 |
| GRSM | Ozark forests | 11 - 24 | 24.5 - 30.5 | 31 - 38 |
| LIRI | Ozark forests | 13.5 - 22.5 | 23.5 - 31.5 | 32 - 35.5 |
| NERI | Ozark forests | 10.5 - 22 | 22.5 - 29 | 29.5 - 35.5 |
| OZAR | Ozark forests | 19.5 - 27.5 | 28 - 35 | 36 - 40 |
| SHEN | Ozark forests | 12.5 - 22.5 | 23 - 30.5 | 31 - 37.5 |
| BITH | SE USA plains | 24.5 - 24.5 | 29.5 - 36 | 36.5 - 36.5 |
| CHAT | SE USA plains | 16 - 25.5 | 26 - 33 | 33.5 - 38.5 |
| CONG | SE USA plains | 18.5 - 27 | 27.5 - 34.5 | 35 - 37.5 |
| MACA | SE USA plains | 16 - 26 | 26.5 - 32.5 | 33 - 38 |
| PRWI | SE USA plains | 19 - 23 | 23.5 - 30 | 30.5 - 35 |
| CAVE | Temperate Sierras | 17 - 25.5 | 26 - 35.5 | 36 - 41 |
| GUMO | Temperate Sierras | 14.5 - 23 | 24 - 31.5 | 32 - 37 |
| CHIR | S semi-arid highlands | 20 - 24 | 24.5 - 31.5 | 32 - 36 |
| SAGU | S semi-arid highlands | 23 - 31 | 31.5 - 38 | 38.5 - 43.5 |
| CHIS | Mediterranean CA | 16.5 - 22 | 22.5 - 28 | 28.5 - 35.5 |
| GOGA | Mediterranean CA | 13.5 - 18 | 18.5 - 25 | 25.5 - 39 |
| PINN | Mediterranean CA | 16 - 23.5 | 24 - 33.5 | 34 - 39.5 |
| PORE | Mediterranean CA | 13.5 - 18.5 | 19 - 26.5 | 27 - 39.5 |
| SAMO | Mediterranean CA | 14.5 - 22 | 22.5 - 31 | 31.5 - 41.5 |
| REDW | Marine west coast forest | 12 - 19 | 19.5 - 25.5 | 26 - 34.5 |

**APPENDIX B.** Sample sizes based on temperature and precipitation groupings.

**Supplementary Table B1** Sample sizes for each group based on daily temperature and precipitation at the visitor center, by ecoregion. Sample sizes represent the total Photo-user-days (only counting one photo per user per day, within a 10-meter radius) in each category.

| **Ecoregion** | **Total *n*** | **Cold days** | **Average days** | **Hot days** | **No precip.** | **Precip.** |
| --- | --- | --- | --- | --- | --- | --- |
| Warm deserts | 25,784 | 4,543 | 17,623 | 3,618 | 24,623 | 1,161 |
| Southern semi-arid highlands | 1,258 | 234 | 823 | 201 | 1,024 | 234 |
| Tropical wet forests | 2,157 | 448 | 1,485 | 224 | 1,077 | 1,080 |
| Southeastern USA plains | 1,391 | 201 | 985 | 205 | 957 | 434 |
| Temperate Sierras | 797 | 110 | 573 | 114 | 697 | 100 |
| Mississippi alluvial and southeast USA coastal plains | 18,337 | 2,832 | 12,969 | 2,536 | 13,237 | 5,100 |
| Cold deserts | 86,804 | 13,871 | 59,961 | 12,972 | 72,301 | 14,503 |
| Ozark, Ouachita-Appalachian forests | 17,830 | 2,506 | 12,638 | 2,686 | 11,017 | 6,813 |
| Great plains | 24,901 | 3,708 | 17,550 | 3,643 | 18,221 | 6,680 |
| Mixed wood plains | 14,228 | 2,334 | 9,838 | 2,056 | 9,589 | 4,639 |
| Northern forest | 6,035 | 905 | 4,369 | 761 | 4,196 | 1,839 |
| Northwest forested mountains | 209,173 | 32,764 | 148,875 | 27,534 | 175,730 | 33,443 |
| Mediterranean California | 76,508 | 11,564 | 53,691 | 11,253 | 74,483 | 2,025 |
| Marine west coast forest | 3,858 | 577 | 2,728 | 553 | 3,273 | 585 |

**APPENDIX C.** Full statistical results associated with Figure 2.

**Supplementary Table C1.** Welch’s ANOVA comparing distributions on hot, cold, and average days by ecoregion.

|  | **Mean: Cold days** | **Mean: Avg. days** | **Mean: Hot days** | **Welch’s ANOVA p-value** | **Cold-Avg Games-Howell p-value** | **Hot-Avg Games-Howell p-value** | **Cold-Avg Cohen’s d** | **Hot-Avg Cohen’s d** |
| --- | --- | --- | --- | --- | --- | --- | --- | --- |
| **ELEVATION** | | | | | | |  |  |
| Warm deserts | 733.296 | 724.952 | 694.836 | **0.003** | 0.614 | **0.007** | 0.015 | -0.053 |
| S semi-arid highlands | 1035.536 | 1077.736 | 1270.182 | **0.000** | 0.386 | **0.000** | -0.094 | 0.398 |
| Tropical wet forests | 1.002 | 1.138 | 1.106 | **0.012** | **0.008** | 0.888 | -0.150 | -0.034 |
| SE USA plains | 203.030 | 196.348 | 200.127 | 0.581 | 0.596 | 0.851 | 0.075 | 0.042 |
| Temperate Sierras | 1581.121 | 1498.523 | 1577.712 | **0.032** | 0.098 | 0.152 | 0.248 | 0.232 |
| MS alluvial/SE coastal plains | 3.958 | 3.687 | 3.522 | 0.074 | 0.177 | 0.507 | 0.038 | -0.024 |
| Cold deserts | 1785.153 | 1833.520 | 1867.240 | **0.000** | **0.000** | **0.000** | -0.095 | 0.068 |
| Ozark forests | 679.837 | 792.198 | 751.373 | **0.000** | **0.000** | **0.000** | -0.229 | -0.081 |
| Great plains | 384.359 | 384.422 | 389.557 | 0.537 | 1.000 | 0.516 | 0.000 | 0.020 |
| Mixed wood plains | 162.345 | 174.157 | 175.764 | **0.000** | **0.000** | 0.864 | -0.092 | 0.012 |
| Northern forest | 208.290 | 211.912 | 210.063 | 0.063 | 0.060 | 0.543 | -0.076 | -0.039 |
| NW forested mountains | 1873.089 | 2019.382 | 2040.450 | **0.000** | **0.000** | **0.000** | -0.191 | 0.027 |
| Mediterranean CA | 97.341 | 80.862 | 77.970 | **0.000** | **0.000** | 0.082 | 0.119 | -0.021 |
| Marine westcoast forest | 96.088 | 99.629 | 85.508 | **0.039** | 0.826 | **0.030** | -0.028 | -0.113 |
| **DISTANCE TO ROADS** | | | | | | | | |
| Warm deserts | 102.386 | 82.013 | 69.919 | **0.000** | **0.000** | **0.034** | 0.072 | -0.045 |
| S semi-arid highlands | 16.543 | 27.298 | 31.775 | **0.002** | **0.003** | 0.802 | -0.182 | 0.063 |
| Tropical wet forests | 66.412 | 126.504 | 188.014 | **0.000** | **0.002** | 0.147 | -0.153 | 0.145 |
| SE USA plains | 7.036 | 10.141 | 7.323 | **0.004** | **0.011** | 0.056 | -0.178 | -0.158 |
| Temperate Sierras | 175.881 | 157.162 | 195.439 | 0.438 | 0.820 | 0.447 | 0.066 | 0.134 |
| MS alluvial/SE coastal plains | 351.900 | 127.568 | 124.043 | **0.000** | **0.000** | 0.947 | 0.273 | -0.006 |
| Cold deserts | 81.744 | 72.175 | 62.714 | **0.000** | **0.010** | **0.010** | 0.027 | -0.027 |
| Ozark forests | 15.999 | 17.578 | 17.019 | **0.035** | **0.028** | 0.650 | -0.048 | -0.017 |
| Great plains | 9.417 | 9.438 | 8.630 | 0.523 | 1.000 | 0.683 | 0.000 | -0.008 |
| Mixed wood plains | 41.461 | 67.961 | 26.607 | **0.000** | **0.001** | **0.000** | -0.071 | -0.116 |
| Northern forest | 90.429 | 75.825 | 67.186 | 0.550 | 0.734 | 0.809 | 0.033 | -0.021 |
| NW forested mountains | 55.205 | 74.799 | 78.412 | **0.000** | **0.000** | 0.055 | -0.075 | 0.013 |
| Mediterranean CA | 25.481 | 24.816 | 31.156 | **0.000** | 0.809 | **0.000** | 0.006 | 0.057 |
| Marine westcoast forest | 15.791 | 15.232 | 13.899 | 0.184 | 0.840 | 0.245 | 0.026 | -0.065 |
| **DISTANCE TO WATERBODIES** | | | | | | | | |
| Warm deserts | 3995.644 | 3646.861 | 3572.485 | 0.068 | 0.079 | 0.890 | 0.038 | -0.008 |
| S semi-arid highlands | 353.658 | 349.821 | 380.113 | 0.876 | 0.995 | 0.864 | 0.007 | 0.051 |
| Tropical wet forests | 295.816 | 324.898 | 335.397 | 0.645 | 0.669 | 0.973 | -0.045 | 0.016 |
| SE USA plains | 137.589 | 151.670 | 123.603 | 0.267 | 0.819 | 0.248 | -0.051 | -0.107 |
| Temperate Sierras | 6333.899 | 5568.635 | 6651.411 | **0.000** | **0.061** | **0.000** | 0.260 | 0.382 |
| MS alluvial/SE coastal plains | 76.388 | 72.428 | 72.513 | 0.199 | 0.178 | 0.999 | 0.036 | 0.001 |
| Cold deserts | 886.973 | 950.799 | 958.598 | **0.000** | **0.000** | 0.910 | -0.033 | 0.004 |
| Ozark forests | 203.251 | 219.231 | 196.554 | **0.002** | 0.095 | **0.004** | -0.045 | -0.064 |
| Great plains | 945.851 | 865.231 | 894.717 | 0.102 | 0.096 | 0.669 | 0.040 | 0.015 |
| Mixed wood plains | 78.081 | 89.865 | 84.375 | **0.000** | **0.000** | 0.163 | -0.091 | -0.042 |
| Northern forest | 57.479 | 57.252 | 51.234 | 0.079 | 0.997 | 0.077 | 0.002 | -0.065 |
| NW forested mountains | 122.748 | 120.821 | 111.335 | **0.000** | 0.291 | **0.000** | 0.009 | -0.044 |
| Mediterranean CA | 97.285 | 78.232 | 74.115 | **0.000** | **0.000** | **0.021** | 0.114 | -0.026 |
| Marine westcoast forest | 216.670 | 223.617 | 221.811 | 0.826 | 0.811 | 0.988 | -0.026 | -0.007 |
| **DISTANCE TO PARKING** | | | | | | | | |
| Warm deserts | 1589.260 | 1052.909 | 1298.883 | **0.000** | 0.066 | **0.000** | 0.122 | 0.061 |
| S semi-arid highlands | 237.017 | 352.973 | 450.655 | **0.004** | 0.148 | **0.007** | -0.151 | 0.095 |
| Tropical wet forests | 409.079 | 730.961 | 753.294 | **0.000** | **0.000** | **0.000** | -0.263 | 0.017 |
| SE USA plains | 469.561 | 597.531 | 416.414 | 0.090 | 0.891 | 0.386 | -0.094 | -0.134 |
| Temperate Sierras | 1160.294 | 515.308 | 672.938 | **0.038** | 0.233 | 0.065 | 0.401 | 0.134 |
| MS alluvial/SE coastal plains | 788.146 | 560.526 | 550.021 | **0.000** | **0.000** | **0.000** | 0.160 | -0.009 |
| Cold deserts | 706.858 | 536.263 | 440.329 | **0.000** | **0.000** | **0.000** | 0.113 | -0.071 |
| Ozark forests | 551.742 | 497.657 | 499.397 | 0.199 | 0.338 | 0.176 | 0.040 | 0.001 |
| Great plains | 321.116 | 282.146 | 427.325 | 0.148 | 0.490 | 0.809 | 0.013 | 0.045 |
| Mixed wood plains | 250.047 | 504.818 | 276.349 | **0.000** | 0.819 | **0.000** | -0.116 | -0.103 |
| Northern forest | 619.239 | 777.490 | 761.856 | **0.046** | 0.257 | **0.035** | -0.112 | -0.011 |
| NW forested mountains | 343.501 | 433.998 | 416.899 | **0.000** | **0.000** | **0.000** | -0.082 | -0.015 |
| Mediterranean CA | 112.927 | 97.880 | 101.228 | **0.000** | **0.002** | **0.000** | 0.060 | 0.013 |
| Marine westcoast forest | 237.570 | 252.599 | 318.898 | **0.003** | **0.011** | 0.772 | -0.037 | 0.168 |
| **DISTANCE TO BUILDINGS** | | | | | | | | |
| Warm deserts | 569.983 | 434.513 | 466.860 | **0.000** | **0.000** | 0.286 | 0.121 | 0.030 |
| S semi-arid highlands | 292.669 | 418.695 | 465.286 | **0.001** | **0.001** | 0.883 | -0.183 | 0.052 |
| Tropical wet forests | 247.423 | 472.330 | 726.046 | **0.000** | **0.000** | **0.035** | -0.207 | 0.212 |
| SE USA plains | 171.269 | 187.180 | 115.008 | **0.022** | 0.902 | **0.016** | -0.034 | -0.163 |
| Temperate Sierras | 1210.188 | 490.381 | 648.624 | **0.023** | **0.037** | 0.326 | 0.439 | 0.132 |
| MS alluvial/SE coastal plains | 133.107 | 95.219 | 103.223 | **0.000** | **0.000** | 0.125 | 0.167 | 0.041 |
| Cold deserts | 640.364 | 569.305 | 524.969 | **0.000** | **0.000** | **0.000** | 0.058 | -0.038 |
| Ozark forests | 213.028 | 192.754 | 203.610 | 0.181 | 0.192 | 0.635 | 0.038 | 0.020 |
| Great plains | 270.049 | 253.426 | 296.495 | **0.020** | 0.476 | **0.019** | 0.021 | 0.054 |
| Mixed wood plains | 216.194 | 282.283 | 238.964 | **0.000** | **0.000** | **0.012** | -0.096 | -0.063 |
| Northern forest | 582.515 | 617.514 | 519.802 | 0.093 | 0.855 | 0.076 | -0.022 | -0.067 |
| NW forested mountains | 273.514 | 304.436 | 291.331 | **0.000** | **0.000** | **0.000** | -0.056 | -0.024 |
| Mediterranean CA | 623.473 | 539.071 | 512.816 | **0.001** | **0.004** | 0.522 | 0.098 | -0.031 |
| Marine westcoast forest | 576.513 | 489.555 | 453.495 | **0.001** | **0.008** | 0.257 | 0.158 | -0.069 |

**APPENDIX D.** Full statistical results associated with Figure 4.

**Supplementary Table D1.** Welch’s t-tests comparing distributions on days with no precipitation to days with precipitation, by ecoregion.

|  | **Mean: no precipitation** | **Mean: precipitation** | **p-value** | **Cohen’s d** | |
| --- | --- | --- | --- | --- | --- |
| **ELEVATION** | | | | | |
| Warm deserts | 717.235 | 827.427 | **0.000** | -0.197 | |
| S semi-arid highlands | 1083.800 | 1174.308 | **0.018** | -0.189 | |
| Tropical wet forests | 1.053 | 1.160 | **0.007** | -0.117 | |
| SE USA plains | 191.913 | 211.008 | **0.000** | -0.214 | |
| Temperate Sierras | 1495.471 | 1700.929 | **0.000** | -0.598 | |
| MS alluvial/SE coastal plains | 3.733 | 3.636 | 0.409 | 0.014 | |
| Cold deserts | 1824.627 | 1861.756 | **0.000** | -0.074 | |
| Ozark forests | 752.506 | 798.958 | **0.000** | -0.094 | |
| Great plains | 395.204 | 357.775 | **0.000** | 0.145 | |
| Mixed wood plains | 175.480 | 166.193 | **0.000** | 0.072 | |
| Northern forest | 210.761 | 211.990 | 0.352 | -0.026 | |
| NW forested mountains | 2001.266 | 1988.599 | **0.004** | 0.016 | |
| Mediterranean CA | 83.423 | 64.694 | **0.000** | 0.136 | |
| Marine westcoast forest | 100.349 | 78.758 | **0.000** | 0.172 | |
| **DISTANCE TO ROADS** | | | | | |
| Warm deserts | 82.099 | 122.218 | **0.003** | -0.144 | |
| S semi-arid highlands | 23.728 | 36.009 | 0.061 | -0.186 | |
| Tropical wet forests | 124.465 | 116.369 | 0.640 | 0.020 | |
| SE USA plains | 9.391 | 9.025 | 0.709 | 0.021 | |
| Temperate Sierras | 156.452 | 226.336 | **0.038** | -0.244 | |
| MS alluvial/SE coastal plains | 174.310 | 129.067 | **0.000** | 0.057 | |
| Cold deserts | 74.406 | 61.743 | **0.000** | 0.036 | |
| Ozark forests | 17.037 | 17.650 | 0.213 | -0.019 | |
| Great plains | 10.158 | 7.022 | **0.000** | 0.033 | |
| Mixed wood plains | 66.157 | 40.028 | **0.000** | 0.075 | |
| Northern forest | 64.878 | 102.224 | **0.019** | -0.088 | |
| NW forested mountains | 75.782 | 53.416 | **0.000** | 0.087 | |
| Mediterranean CA | 25.698 | 31.469 | **0.038** | -0.052 | |
| Marine westcoast forest | 15.274 | 14.288 | 0.261 | 0.047 | |
| **DISTANCE TO WATERBODIES** | | | | | |
| Warm deserts | 3490.725 | 8091.293 | **0.000** | -0.505 | |
| S semi-arid highlands | 329.268 | 469.620 | **0.013** | -0.240 | |
| Tropical wet forests | 287.111 | 352.694 | **0.018** | -0.102 | |
| SE USA plains | 139.511 | 158.703 | 0.231 | -0.071 | |
| Temperate Sierras | 5934.186 | 5096.904 | **0.002** | 0.287 | |
| MS alluvial/SE coastal plains | 74.442 | 69.441 | **0.004** | 0.046 | |
| Cold deserts | 960.319 | 849.269 | **0.000** | 0.058 | |
| Ozark forests | 220.968 | 201.604 | **0.000** | 0.055 | |
| Great plains | 960.132 | 667.203 | **0.000** | 0.149 | |
| Mixed wood plains | 92.831 | 75.372 | **0.000** | 0.136 | |
| Northern forest | 55.680 | 58.462 | 0.275 | -0.030 | |
| NW forested mountains | 120.075 | 118.821 | 0.309 | 0.006 | |
| Mediterranean CA | 80.783 | 70.344 | **0.010** | 0.063 | |
| Marine westcoast forest | 216.552 | 254.583 | **0.001** | -0.145 | |
| **DISTANCE TO PARKING** | | | | | |
| Warm deserts | 1138.106 | 2111.281 | **0.000** | | -0.214 |
| S semi-arid highlands | 314.737 | 488.248 | 0.121 | | -0.184 |
| Tropical wet forests | 588.471 | 744.166 | **0.003** | | -0.128 |
| SE USA plains | 544.165 | 570.388 | 0.736 | | -0.020 |
| Temperate Sierras | 604.803 | 780.709 | 0.191 | | -0.113 |
| MS alluvial/SE coastal plains | 635.943 | 485.954 | **0.000** | | 0.109 |
| Cold deserts | 552.066 | 534.838 | 0.159 | | 0.012 |
| Ozark forests | 528.246 | 468.773 | **0.003** | | 0.045 |
| Great plains | 365.037 | 156.851 | **0.000** | | 0.063 |
| Mixed wood plains | 491.480 | 302.948 | **0.000** | | 0.089 |
| Northern forest | 781.969 | 689.876 | **0.041** | | 0.064 |
| NW forested mountains | 424.566 | 380.824 | **0.000** | | 0.041 |
| Mediterranean CA | 100.455 | 107.517 | 0.257 | | -0.028 |
| Marine westcoast forest | 273.715 | 182.306 | **0.000** | | 0.222 |
| **DISTANCE TO BUILDINGS** | | | | | |
| Warm deserts | 459.378 | 538.059 | **0.030** | -0.070 | |
| S semi-arid highlands | 370.259 | 544.649 | 0.065 | -0.211 | |
| Tropical wet forests | 408.153 | 495.657 | 0.073 | -0.077 | |
| SE USA plains | 175.735 | 170.957 | 0.857 | 0.011 | |
| Temperate Sierras | 582.249 | 822.247 | 0.095 | -0.151 | |
| MS alluvial/SE coastal plains | 104.157 | 97.039 | **0.042** | 0.032 | |
| Cold deserts | 580.556 | 541.520 | **0.000** | 0.032 | |
| Ozark forests | 212.291 | 172.898 | **0.000** | 0.073 | |
| Great plains | 297.094 | 167.027 | **0.000** | 0.164 | |
| Mixed wood plains | 299.900 | 193.417 | **0.000** | 0.157 | |
| Northern forest | 624.292 | 544.391 | 0.063 | 0.052 | |
| NW forested mountains | 308.311 | 242.990 | **0.000** | 0.120 | |
| Mediterranean CA | 542.751 | 732.033 | **0.008** | -0.223 | |
| Marine westcoast forest | 494.896 | 511.353 | 0.492 | -0.030 | |

**APPENDIX E.** Study sites by ecoregion.

**Supplementary Table E1.** The NPS units included in this paper, by ecoregion.

| **Ecoregion** | **Number of units** | **NPS Units** |
| --- | --- | --- |
| Northern forest | 6 | Apostle Islands NL, Isle Royale NP, Pictured Rocks NL, Saint Croix NSR, Upper Delaware S&RR, Voyageurs NP |
| Northwest forested mountains | 20 | Bandelier NM, Crater Lake NP, Curecanti NRA, Glacier NP, Grand Teton NP, John Day Fossil Beds NM, Kings Canyon NP, Lava Beds NM, Lake Chelan NRA, Lassen Volcanic NP, Mount Rainier NP, North Cascades NP, Olympic NP, Ross Lake NRA, Rocky Mountain NP, Sequoia NP, Whiskeytown NRA, Wind Cave NP, Yellowstone NP, Yosemite NP |
| Marine west coast forest | 1 | Redwood NP |
| Eastern temperate forest: Mixed wood plains | 4 | Acadia NP, Cuyahoga Valley NP, Indiana Dunes NP, Sleeping Bear Dunes NL |
| Eastern temperate forest: Southeastern USA plains | 5 | Big Thicket NPRES, Chattahoochee River NRA, Congaree NP, Mammoth Cave NP, Prince William Forest Park |
| Eastern temperate forest: Ozark, Ouachita-Appalachian forests | 10 | Big South Fork NRRA, Buffalo NR, Cumberland Cap NHP, Delaware Water Gap NRA, Gauley River NRA, Great Smoky Mountains NP, Little River Canyon NPRES, New River Gorge NR, Ozark NSR, Shenandoah NP |
| Eastern temperate forest: Mississippi Alluvial and Southeast USA coastal plains | 11 | Assateague Island NS, Cape Cod NS, Cape Hatteras NS, Cape Lookout NS, Canaveral NS, Cumberland Island NS, Fire Island NS, Gateway NRA, Gulf Island NS, Jean Lafitte NHP & PRES, Timucuan EHP |
| Great plains | 10 | Badlands NP, Bighorn Canyon NRA, Lake Meredith NRA, Mississippi NRRA, Missouri NRR, Niobrara NSR, Padre Island NS, Sand Creek Massacre NHS, Tallgrass Prairie NPRES, Theodore Roosevelt NP |
| North American deserts: cold deserts | 21 | Arches NP, Black Canyon of the Gunnison NP, Bryce Canyon NP, Canyon de Chelly NM, Canyonlands NP, Capitol Reef NP, Chaco Culture NHP, City of Rocks NRES, Colorado NM, Craters of the Moon NM & PRES, Dinosaur NM, El Malpais NM, Glen Canyon NRA, Great Basin NP, Grand Canyon NP, Great Sand Dunes NP & PRES, Lake Roosevelt NRA, Mesa Verde NP, Petrified Forest NP, Wupatki NM, Zion NP |
| North American deserts: warm deserts | 9 | Amistad NRA, Big Bend NP, Death Valley NP, Joshua Tree NP, Lake Mead NRA, Mojave NPRES, Organ Pipe Cactus NM, Rio Grande W&SR, White Sands NM |
| Mediterranean California | 5 | Channel Islands NP, Golden Gate NRA, Pinnacles NP, Point Reyes NS, Santa Monica Mountains NRA |
| Southern semi-arid highlands | 2 | Chiricahua NM, Saguaro NP |
| Temperate Sierras | 2 | Carlsbad Caverns NP, Guadalupe Mountains NP |
| Tropical wet forests | 4 | Big Cypress NPRES, Biscayne NP, Dry Tortugas NP, Everglades NP |
| NP = National Park, NM = National Monument, NRA = National Recreation Area, NS = National Seashore, NHP = National Historical Park, NL = National Lakeshore, NSR = National Scenic River, S&RR = Scenic & Recreational River, NPRES = National Preservation, NR = National River, NRRA = National River & Recreation Area, EHP = Ecological & Historic Preserve, NHS = National Historic Site, W&SR = Wild & Scenic River | | |

**APPENDIX F.** Sample sizes for each study site.

**Supplementary Table F1.** The number of Flickr data points in each study site between May – September, 2006 – 2018. Numbers represent only one post per user, per day, within a 10-meter radius.

| **Code** | **Park Name** | ***n*** | **Code** | **Park Name** | ***n*** |
| --- | --- | --- | --- | --- | --- |
| ACAD | Acadia National Park | 8,101 | GUMO | Guadalupe Mountains National Park | 322 |
| AMIS | Amistad National Recreation Area | 32 | INDU | Indiana Dunes National Lakeshore | 1,372 |
| APIS | Apostle Islands National Lakeshore | 355 | ISRO | Isle Royale National Park | 1,183 |
| ARCH | Arches National Park | 9,020 | JELA | Jean Lafitte National Historical Park and Preserve | 313 |
| ASIS | Assateague Island National Seashore | 1,532 | JODA | John Day Fossil Beds National Monument | 1,151 |
| BADL | Badlands National Park | 4,416 | JOTR | Joshua Tree National Park | 4,552 |
| BAND | Bandelier National Monument | 974 | KICA | Kings Canyon National Park | 7,770 |
| BIBE | Big Bend National Park | 1,688 | LABE | Lava Beds National Monument | 684 |
| BICA | Bighorn Canyon National Recreation Area | 153 | LACH | Lake Chelan National Recreation Area | 313 |
| BICY | Big Cypress National Preserve | 492 | LAKE | Lake Mead National Recreation Area | 8,725 |
| BISC | Biscayne National Park | 52 | LAMR | Lake Meredith National Recreation Area | 20 |
| BISO | Big South Fork National River and Recreation Area | 768 | LARO | Lake Roosevelt National Recreation Area | 303 |
| BITH | Big Thicket National Preserve | 32 | LAVO | Lassen Volcanic National Park | 4,340 |
| BLCA | Black Canyon of the Gunnison National Park | 1,289 | LIRI | Little River Canyon National Preserve | 134 |
| BRCA | Bryce Canyon National Park | 10,581 | MACA | Mammoth Cave National Park | 498 |
| BUFF | Buffalo National River | 490 | MEVE | Mesa Verde National Park | 3,272 |
| CACH | Canyon de Chelly National Monument | 992 | MISS | Mississippi National River and Recreation Area | 18,130 |
| CACO | Cape Cod National Seashore | 3,429 | MNRR | Missouri National Recreation River | 132 |
| CAHA | Cape Hatteras National Seashore | 2,352 | MOJA | Mojave National Preserve | 1,526 |
| CALO | Cape Lookout National Seashore | 201 | MORA | Mount Rainier National Park | 17,415 |
| CANA | Canaveral National Seashore | 341 | NERI | New River Gorge National River | 1,385 |
| CANY | Canyonlands National Park | 4,540 | NIOB | Niobrara National Scenic River | 72 |
| CARE | Capitol Reef National Park | 3,394 | NOCA | North Cascades National Park | 1,880 |
| CAVE | Carlsbad Caverns National Park | 475 | OLYM | Olympic National Park | 12,365 |
| CHAT | Chattahoochee River National Recreation Area | 597 | ORPI | Organ Pipe Cactus National Monument | 216 |
| CHCU | Chaco Culture National Historical Park | 963 | OZAR | Ozark National Scenic Riverway | 316 |
| CHIR | Chiricahua National Monument | 266 | PAIS | Padre Island National Seashore | 141 |
| CHIS | Channel Islands National Park | 1,331 | PEFO | Petrified Forest National Park | 2,836 |
| CIRO | City of Rocks National Reserve | 265 | PINN | Pinnacles National Park | 986 |
| **Code** | **Park Name** | ***n*** | **Code** | **Park Name** | ***n*** |
| COLM | Colorado National Monument | 1,184 | PIRO | Pictured Rocks National Lakeshore | 1,836 |
| CONG | Congaree National Park | 154 | PORE | Point Reyes National Seashore | 6,259 |
| CRLA | Crater Lake National Park | 4,558 | PRWI | Prince William Forest Park | 110 |
| CRMO | Craters of the Moon National Monument | 1,110 | REDW | Redwood National Park | 3,858 |
| CUGA | Cumberland Gap National Historical Park | 231 | RIGR | Rio Grande Wild and Scenic River | 240 |
| CUIS | Cumberland Island National Seashore | 309 | ROLA | Ross Lake National Recreation Area | 1,951 |
| CURE | Curecanti National Recreation Area | 618 | ROMO | Rocky Mountain National Park | 15,152 |
| CUVA | Cuyahoga Valley National Park | 2,523 | SACN | Saint Croix National Scenic Riverway | 335 |
| DEVA | Death Valley National Park | 7,671 | SAGU | Saguaro National Park | 992 |
| DEWA | Delaware Water Gap National Recreation Area | 1,721 | SAMO | Santa Monica Mountains National Recreation Area | 15,385 |
| DINO | Dinosaur National Monument | 1,258 | SAND | Sand Creek Massacre National Historic Site | 37 |
| DRTO | Dry Tortugas National Park | 0 | SEQU | Sequoia National Park | 8,724 |
| ELMA | El Malpais National Monument | 198 | SHEN | Shenandoah National Park | 4,423 |
| EVER | Everglades National Park | 1,613 | SLBE | Sleeping Bear Dunes National Lakeshore | 2,232 |
| FIIS | Fire Island National Seashore | 2,447 | TAPR | Tallgrass Prairie National Preserve | 276 |
| GARI | Gauley River National Recreation Area | 21 | THRO | Theodore Roosevelt National Park | 1,524 |
| GATE | Gateway National Recreation Area | 3,899 | TIMU | Timucuan Ecological and Historic Preserve | 457 |
| GLAC | Glacier National Park | 16,459 | UPDE | Upper Delaware Scenic and Recreational River | 2,189 |
| GLCA | Glen Canyon National Recreation Area | 5,721 | VOYA | Voyageurs National Park | 137 |
| GOGA | Golden Gate National Recreation Area | 52,547 | WHIS | Whiskeytown-Shasta-Trinity National Recreation Area | 248 |
| GRBA | Great Basin National Park | 674 | WHSA | White Sands National Monument | 1,134 |
| GRCA | Grand Canyon National Park | 26,192 | WICA | Wind Cave National Park | 497 |
| GRSA | Great Sand Dunes National Park and Preserve | 1,918 | WUPA | Wupatki National Monument | 472 |
| GRSM | Great Smoky Mountains National Park | 8,341 | YELL | Yellowstone National Park | 56,850 |
| GRTE | Grand Teton National Park | 15,928 | YOSE | Yosemite National Park | 41,296 |
| GUIS | Gulf Islands National Seashore | 3,057 | ZION | Zion National Park | 10,622 |

**APPENDIX G.** Key-value pairs used to download OpenStreetMap data.

**Supplementary Table G1** Key-value pairs used to download OpenStreetMap data for each category of data used in this analysis.

| **Category** | **Key** | **Value(s)** | **Types of data used** |
| --- | --- | --- | --- |
| **Roads** | highway | motorway, trunk, primary, secondary, tertiary, motorway_link, trunk_link, primary_link, tertiary_link, unclassified, residential, service | lines, polygons |
| **Water** | natural | water, bay, strait, coastline | lines, polygons, multipolygons |
|  | waterway | river |  |
| **Parking** | amenity | parking | polygons, multipolygons |
| **Buildings** | building | (all) | polygons, multipolygons |
